# Cryo-EM analysis of scorpion toxin binding to Ryanodine Receptors reveals subconductance that is abolished by PKA phosphorylation

**DOI:** 10.1126/sciadv.adf4936

**Published:** 2023-05-24

**Authors:** Omid Haji-Ghassemi, Yu Seby Chen, Kellie Woll, Georgina B. Gurrola, Carmen R. Valdivia, Wenxuan Cai, Songhua Li, Hector H. Valdivia, Filip Van Petegem

**Affiliations:** ^1^ Department of Biochemistry and Molecular Biology, Life Sciences Centre, University of British Columbia, Vancouver, BC, Canada.; ^2^ Universidad Nacional Autónoma de México, Departamento de Medicina Molecular y Bioprocesos, Instituto de Biotechnología, Cuaernavaca, Morelos 62271, Mexico.; ^3^ Department of Medicine and Cardiovascular Research Center, School of Medicine and Public Health, University of Wisconsin-Madison, Madison, WI, USA.; ^4^ Department of Cardiology, Changhai Hospital, Naval Medical University, Shanghai, China.

## Abstract

Calcins are peptides from scorpion venom with the unique ability to cross cell membranes, gaining access to intracellular targets. Ryanodine Receptors (RyR) are intracellular ion channels that control release of Ca^2+^ from the endoplasmic and sarcoplasmic reticulum. Calcins target RyRs and induce long-lived subconductance states, whereby single-channel currents are decreased. We used cryo–electron microscopy to reveal the binding and structural effects of imperacalcin, showing that it opens the channel pore and causes large asymmetry throughout the cytosolic assembly of the tetrameric RyR. This also creates multiple extended ion conduction pathways beyond the transmembrane region, resulting in subconductance. Phosphorylation of imperacalcin by protein kinase A prevents its binding to RyR through direct steric hindrance, showing how posttranslational modifications made by the host organism can determine the fate of a natural toxin. The structure provides a direct template for developing calcin analogs that result in full channel block, with potential to treat RyR-related disorders.

## Teaser

Cryo-EM images show how scorpion peptides partially obstruct Ca^2+^ flow through Ryanodine Receptors by forming an imperfect wedge.

## INTRODUCTION

Peptide-based toxins, obtained from venomous species, often target ion channels, rapidly interfering with electrical signals and resulting in paralysis or severe pain. Usually targeting exposed extracellular portions, they hold tremendous potential for therapeutic use, as shown by the use of ziconotide (also known as Prialt) for the treatment of severe pain (*1*). However, as most peptide-based toxins cannot cross cellular membranes, intracellular ion channels are usually unaffected, hampering the development of analogs that could target them for therapeutic use.

**Fig. 1.**
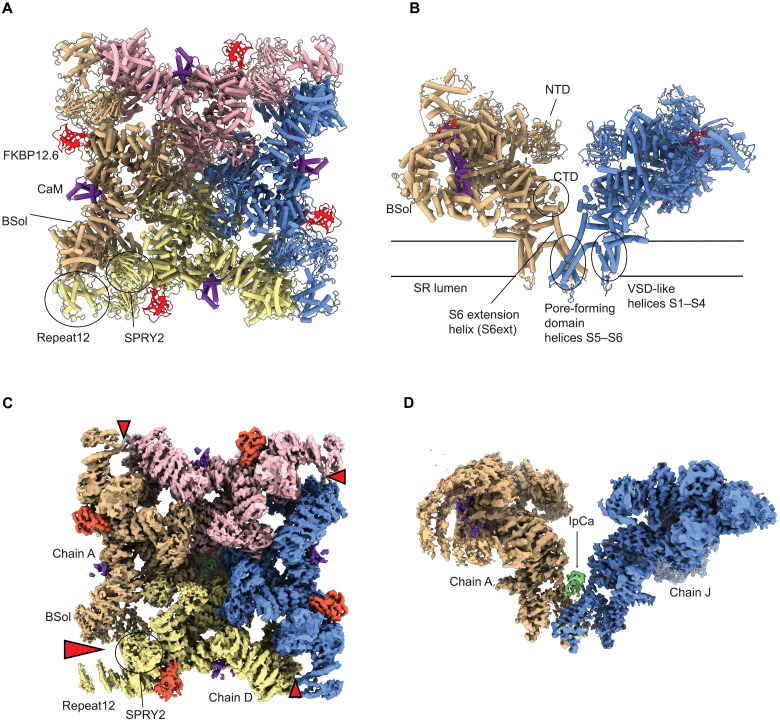
Overall view of RyR1 and its binding to IpCa. (**A**) Top view (from the cytosol facing the SR) and (**B**) side view of rabbit RyR1 bound to FKBP12.6 (red) and calmodulin (CaM) (purple) [Protein Data Bank (PDB) 7TZC]. The four RyR1 subunits are shown in different colors. Select domains are labeled (Bsol, bridging solenoid domain; NTD, N-terminal domain; CTD, C-terminal domain; VSD-like, voltage-sensing like domain). In (B), only two subunits are shown for clarity. (**C**) Top view and (**D**) side view of a cryo-EM map of RyR1 bound to IpCa (this study, dataset 3, global refined structure). The density is colored as in (A) and (B), with density for IpCa shown in green. The red wedges in (C) highlight the asymmetry, with a clear break between chains A and D at the Bsol-SPRY2-Repeat12 interface. For a better view of the asymmetry, see movie S2.

Calcins are a family of scorpion-derived peptides that can penetrate cell membranes and bind to Ryanodine Receptors (RyRs) with high affinity and specificity (*2*, *3*). RyRs form massive (~2.2 MDa) channels that are embedded within the membranes of the endoplasmic or sarcoplasmic reticulum (SR), where they control the release of Ca^2+^. They form homotetrameric assemblies, with the bulk of the protein exposed in the cytosol (Fig. 1, A and B). The human genome encodes three isoforms (RyR1 to RyR3) that are expressed in multiple tissues (*4*). The RyR1 and RyR2 isoforms are particularly abundant in skeletal and cardiac muscle, respectively, where they play a central role in muscle excitation-contraction coupling. They are associated with hundreds of disease mutations that cause malignant hyperthermia, stress-induced arrhythmias, and myopathies and play critical roles in atrial fibrillation, Alzheimer’s progression, and more (*5*). Yet, efficient therapies targeting RyRs directly are lacking.

Imperacalcin (IpCa, also known as imperatoxin-A) is the founding member of the calcin family and was obtained from the African scorpion *Pandinus* imperator (*3*). Since then, other calcins have been found, all bearing ~80 to 90% sequence identity and composed of 33 to 35 amino acid residues distinctively arranged in a globular, cysteine-knotted structure supported by three disulfide bonds (*6*, *7*). Unlike other ion channels targeted by peptide toxins, RyRs are not exposed on the extracellular surface, yet calcins can efficiently reach them by permeating the plasma membrane (*8*, *9*). Upon engaging the RyR, calcins cause long-lived subconductance states, which are readily detected in single-channel recordings as decreased current levels at 20 to 60% of the full conductance (*2*, *10*–*14*). The mechanism by which calcin binding results in subconductance, and how the subconductance level depends on the calcin’s specific amino acid composition, had remain unsolved.

Here, we used cryo–electron microscopy (cryo-EM), RyR mutagenesis, and functional assays to show that IpCa enters through the vestibule of the RyR and binds with high affinity to a site deep in the cytosolic cap, adjacent to the transmembrane region. The binding causes short- and long-range conformational changes, including a widening of the channel pore, the induction of asymmetric breaks, and the formation of extended narrow ion conduction pathways that explain the subconducting state. Protein kinase A (PKA) may effectively neutralize the calcin by adding a phosphate group that results in steric hindrance with the binding site. As calcins directly permeate cell membranes, our structural analysis offers valuable clues to generate calcin variants that can fully block RyRs, with the potential to treat their gain-of-function phenotype in disease.

## RESULTS

### IpCa binds most efficiently to activated channels

**Fig. 2.**
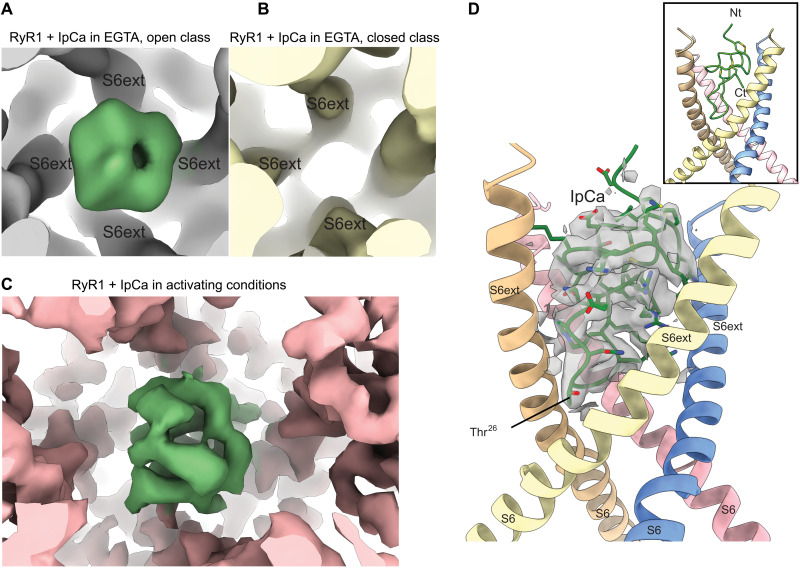
Density for IpCa in different conditions. (**A**) Density of IpCa (green) bound to RyR1 (gray) in the absence of any other activating ligands (open class dataset 1). The positions of the S6ext helices are indicated. IpCa can thus bind to RyR1 in the absence of any activating ligands. (**B**) Corresponding region for the closed class of dataset 1, showing no density for IpCa (RyR1 density in yellow). (**C**) Density figure for IpCa (green) bound to RyR1 (pink) in the presence of activating conditions (Ca^2+^, ATP, caffeine, CaM_1234_, dataset 3, locally refined map). (**D**) Cartoon for the cytosolic extensions of the inner S6 helices (S6ext) with density (green) for IpCa (oxygen atom in red, nitrogen in blue, and carbon in green stick representation). RyR helices are colored by subunit (chain A, wheat; D, yellow; G, pink; J, blue). The inset shows the same view, but without density and showing the IpCa in cartoon form, with side chains for cysteines (sulfur atoms in yellow).

We collected several cryo-EM datasets for rabbit RyR1, purified from skeletal muscle, in the presence of 30 to 40 μM IpCa, under different conditions (datasets 1 to 3; figs. S1 and S2). In multiple classes across datasets, additional density can be observed on the fourfold symmetry axis, adjacent to the transmembrane region (Figs. 1, C and D, and 2). In the absence of any other ligands (dataset 1), we obtained two classes (fig. S1). One corresponds to closed channels that did not show any extra density for IpCa. The other corresponds to an open class with IpCa bound (Fig. 2, A and B). We reasoned that adding a more activating condition would result in a higher fraction of channels bound to IpCa. Addition of only 30 μM free Ca^2+^ resulted in classes that all correspond to open channels and with density for IpCa (dataset 2; fig. S1). Previous cryo-EM studies of rabbit RyR1 under nonactivating conditions, or even in the presence of 30 μM free Ca^2+^, only showed closed channels (*15*). We did not observe any additional density in the known calmodulin (CaM) binding region, which was previously proposed as the IpCa binding site (*16*).

To obtain higher-resolution insights into IpCa binding, we collected a larger dataset for RyR1with IpCa in the presence of a cocktail of activators, including 30 μM free Ca^2+^, 5 mM caffeine, and 8 mM adenosine triphosphate (ATP) (dataset 3). Because IpCa does not occupy the CaM binding site, and apoCaM stimulates channel opening (*17*, *18*), we also added CaM_1234_, a CaM mutant unable to bind Ca^2+^ (*19*). Under these conditions, all classes obtained also revealed only open channels displaying density for IpCa (Figs. 1, C and D, and 2, C and D, and fig. S2). The presence of IpCa on the fourfold symmetry axis results in a break of the fourfold symmetry, and we therefore processed all data without any symmetry imposed.

### IpCa binding induces large asymmetry in RyR1

The overall conformation of IpCa-bound RyR1 is highly asymmetric, with a break of the intersubunit interface between the bridging solenoid (Bsol) of one subunit and the SPRY2 domain of a neighboring subunit for nearly all particles (Fig. 1C and fig. S2). Whereas asymmetric particles can sometimes be seen for RyR1 in the absence of IpCa, they only represent a small fraction (~10 to 15%) and with less pronounced breaks, as shown by a similar dataset that does not have any IpCa bound (fig. S3).

Although processed without imposed symmetry, the density for IpCa appeared to be twofold symmetry averaged, with two distinct conformations that can be superposed onto one another with a 180° rotation (fig. S4A). At this level, the particle alignment is dominated by the asymmetric cap and not the relatively small IpCa. This indicates that the precise location of the asymmetric break can occur in one of two locations relative to the orientation of IpCa. Further local refinement resulted in only one predominant conformation (see Materials and Methods for a full explanation). Both scenarios allowed us to unambiguously place previous nuclear magnetic resonance (NMR) structures for calcins (*6*, *7*) in the map (fig. S4). Calcins contain a loop (residues 22 to 30 in IpCa) that has higher flexibility compared to the rest of the peptide. We found that the loop conformation, as previously published for an NMR structure of maurocalcin, gave the best initial fit to the density (fig. S4D), suggesting that the loop in IpCa adjusts to the binding site in RyR1, rendering it more similar to maurocalcin. These initial models were then further refined against the experimental maps.

### The IpCa binding site is formed by the C-terminal S6 extension helices

**Fig. 3.**
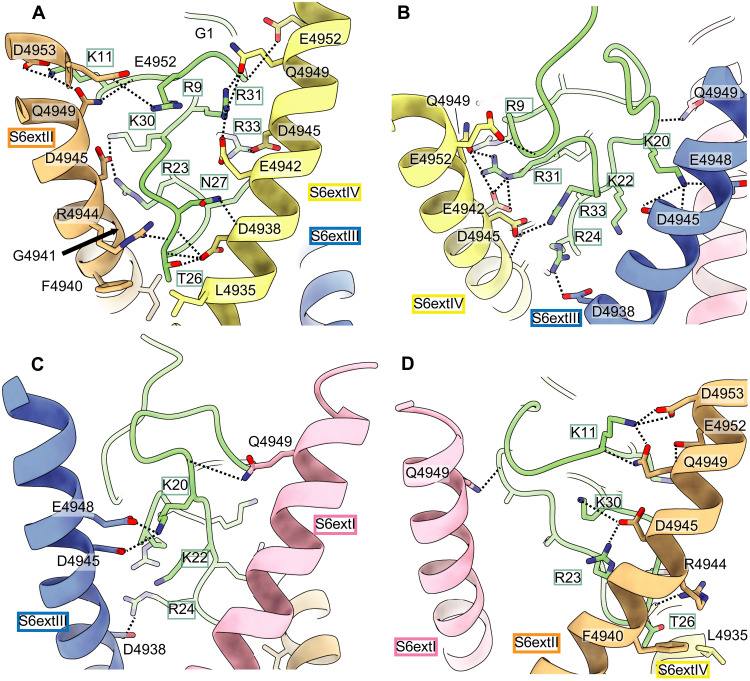
Interactions between IpCa and the S6ext helices of RyR1. (**A** to **D**) Shown are four different views of IpCa (green) and its interactions with residues in the S6ext helices of RyR1. In all panels, the S6ext helices of the four different subunits are colored according to Fig. 1. Dotted lines correspond to putative hydrogen bonds and salt bridges (see table S1 for a complete list). Boxed residues correspond to IpCa, and all other labels correspond to RyR1 residues.

The detailed binding interface between IpCa and RyR1 is shown in Fig. 3, and putative interacting pairs of residues are shown in table S1. IpCa enters the vestibule of the channel and binds deep at the cytosolic side, adjacent to the transmembrane region. All RyR1 residues involved in binding are located in the cytosolic extensions of the inner S6 helices (S6ext) that line the permeation pathway. Equivalent residues of all four RyR1 subunits make different interactions with IpCa. The most extensive interface includes the side of IpCa that contains the 22–30 loop. It mostly consists of various salt bridges between negatively charged RyR1 residues and positively charged IpCa residues, explaining the high conservation of positive charges within all calcins (fig. S5). However, substitutions of positive residues, albeit rare, do occur naturally among calcins, and our data here explain how those changes impinge on their potencies (fig. S5). For example, Lys^20^ in IpCa is near multiple negatively charged residues in RyR1 but is replaced by Ser in urocalcin, which has the weakest apparent affinity of all calcins tested to date (*2*). At the “bottom” tip of the 22–30 loop in IpCa, Thr^26^ is located in a small pocket formed by two adjacent S6ext helices from neighboring subunits, making extensive van der Waals interactions and a possible H-bond with Asp^4938^. Comparing the sequences of opicalcin1 and opicalcin2, which differ ~10-fold in potency, the only change is replacement of opicalcin1 Thr^26^ with Ala^26^ in opicalcin2 (fig. S5). The reduced potency for opicalcin2 is thus likely the result of reduced van der Waals interactions and loss of a H-bond.

The structure is also in agreement with previous mutagenesis studies of calcins. For example, Arg^23^ is conserved in all calcins and forms a likely salt bridge with RyR1 residue Asp^4945^. The IpCa R23E mutation was previously shown to drastically reduce potency (*12*). Another study found a general importance of multiple charged IpCa residues to form a subconductance state in RyR1, with the largest effects found for substitutions in the 22–30 loop (*20*), which forms the most extensive interactions with RyR1. Thus, the structure appears to be in general agreement with known potencies of natural and engineered calcins.

**Fig. 4.**
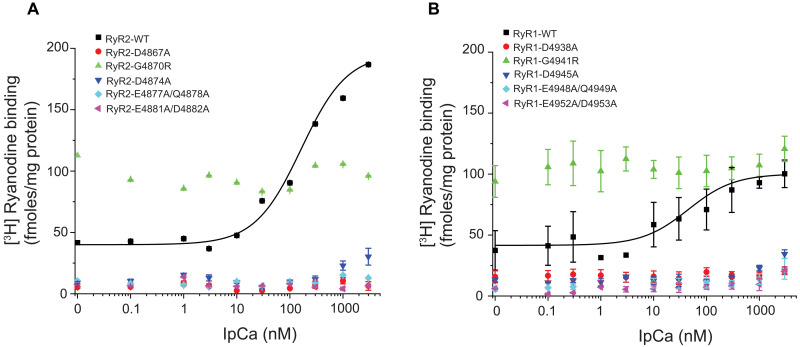
[^3^H] Ryanodine binding experiments. [^3^H] ryanodine binding to wild-type (WT) and mutant RyR2 (**A**) and RyR1 (**B**) as a function of IpCa concentration. IpCa increases the binding of [^3^H] ryanodine by increasing channel Po without interacting with the ryanodine binding site. [^3^H] Ryanodine binding was performed at a fixed [Ca^2+^] of 100 nM for RyR2 and 10 μM for RyR1. Same colors were assigned in both panels to represent the equivalent mutations in RyR1 and RyR2. Data points are the mean of three to five experiments.

On the RyR side, all residues involved in the interaction are conserved among all three mammalian isoforms, indicating that IpCa is likely to engage all isoforms in similar fashion. To verify the involvement of these residues, we performed [^3^H] ryanodine binding assays, which report on the open probability (Po) of RyRs (Fig. 4 and fig. S6). Given that the IpCa binding site is conserved among all three RyR isoforms, we also included RyR2 in the study, as recombinant RyR2 yielded higher [^3^H] ryanodine binding signals than recombinant RyR1 (fig. S6, A to C). As previously shown and in agreement with the structures, the addition of IpCa results in an increase of [^3^H] ryanodine binding to RyR1 at all Ca^2+^ concentrations, in agreement with the ability of IpCa to shift the population toward channels with an open pore. IpCa also increases [^3^H] ryanodine binding to RyR2 at low Ca^2+^ but decreases the maximum amount of [^3^H] ryanodine binding at higher Ca^2+^ concentrations (fig. S6C). The reasons for the latter observation are not clear but suggest that the precise structural perturbations caused by IpCa differ between RyR1 and RyR2. Regardless, mutations in the binding site can decrease or abolish the activating effect of IpCa on [^3^H] ryanodine binding (Fig. 4), thus confirming the importance of this region. Some mutations, most notably G4870R (RyR2) and G4941R (RyR1), inherently enhance [^3^H] ryanodine binding. The S6ext helices undergo substantial conformational changes during normal channel gating, and an inherent effect of some mutations in this area is therefore not unexpected. These mutant channels still appear functional, as they still respond to Ca^2+^ (fig. S6, D to F). However, IpCa is not able to further enhance Po, likely a result from lack of binding to the mutant channels. The fact that most mutations in the S6ext region completely abolish the effect of IpCa also argues against the proposal that the distant CaM binding region forms the IpCa binding site (*16*).

### Mechanism for channel opening and asymmetry caused by IpCa

**Fig. 5.**
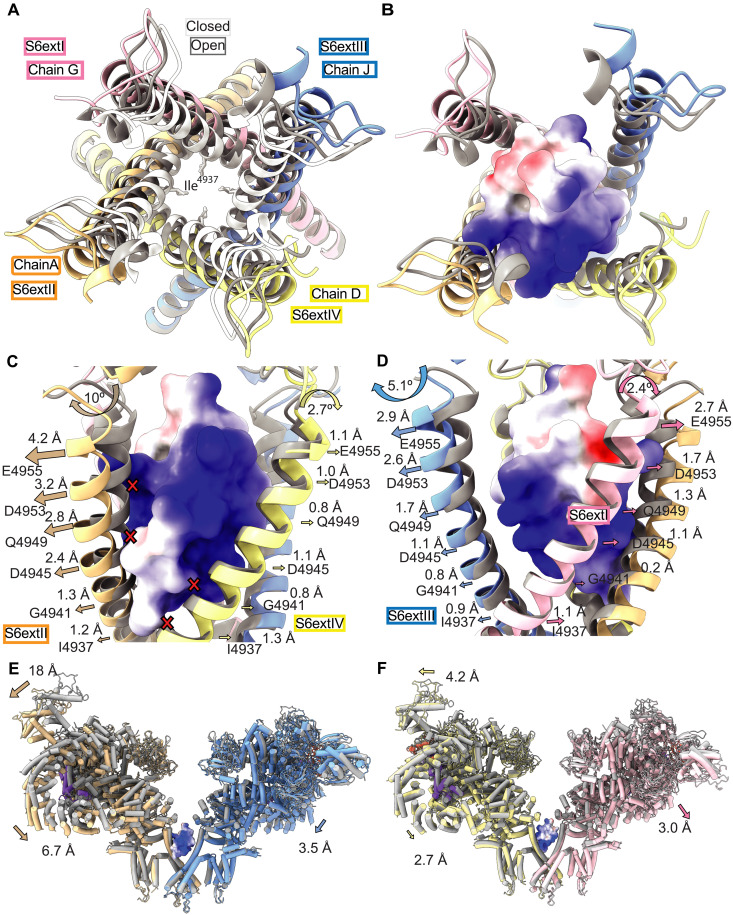
Asymmetric movements induced by IpCa. (**A**) Top view (facing the SR membrane) of the S6ext helices of closed RyR1 (PDB: 7TZC, white cartoon), open RyR1 without IpCa bound (this study, dataset 4, gray) and IpCa-bound RyR1 (dataset 3, locally refined model, colors). The position of Ile^4937^ in the closed structure is indicated for reference. The S6ext helices gradually splay outwards going from closed to open-unbound and open-bound. (**B**) Top view of the open RyR1 without (gray) and with IpCa bound (colors). IpCa is shown in electrostatic surface representation (positive charge in blue, negative charge in red). (**C** and **D**) Side views of the same superposition as in (B), showing the displacement of the Cα atoms at various points along the S6ext helices. Changes in tilt angle are also shown. The largest shifts and tilts are observed for chains A (orange) and J (blue). The red “x” signs indicate positions with major clashes. (**E**) Comparison of open RyR1 model (gray) without bound IpCa (dataset 4) to a globally refined map (3.7 Å, dataset 3) of RyR1 in the presence of IpCa and activating ligands (wheat, chain A; blue, chain J). (**F**) Same as in (E), but showing chains D (yellow) and G (pink).

A superposition of the IpCa-bound structure with closed RyR1 structures straightforwardly reveals the mechanism of channel opening (fig. S7 and Fig. 5A). RyR channel opening results from bending of the inner S6 helices, which also results in a movement of the cytosolic extension (S6ext) of these helices. In the closed state, the S6ext helices are too close to allow for binding of IpCa (fig. S7). Together with the absence of any IpCa density in closed channels, this suggests that IpCa indirectly promotes channel opening by selecting the open state. However, short-lived transient states with IpCa bound to closed channels in a different conformation cannot be ruled out.

Comparison with other open RyR1 channel structures, not bound to IpCa, shows that IpCa causes an additional dilation of the S6ext helices, which need to move further outward to avoid steric clashes with IpCa. To obtain a good reference structure, we collected and solved a structure of RyR1 in the same conditions as the IpCa-bound dataset but lacking any bound calcin (dataset 4; see section below), which also displayed an open pore. Superposition based on the pore-forming domains shows that the S6ext helices move laterally to accommodate IpCa but to different extents (Fig. 5, A to D, and movie S1). These movements can be described as bending of the S6ext helices with a hinge point near Gly^4941^. The degree of bending is the largest for chain A (~10°) (Fig. 5, C and D). At the level of the S6ext helices, the internal diameter thus widens from closed RyR1 to open RyR1, to IpCa-bound RyR1 (Fig. 5A).

This asymmetric movement of the S6ext helices brings about marked asymmetric conformational changes throughout the large cytosolic cap (Fig. 5, E and F). In the global refined structure, the largest movement of S6ext is also observed for chain A (~4.2 Å). As this movement is primarily a tilt, movements far away from the hinge point are larger in magnitude. The S6ext is followed in sequence by the C-terminal domain (CTD), which is grasped by a “Thumb and Forefinger” domain, in turn linked to the central solenoid (Csol) (fig. S8A). There are also direct interactions between the CTD and the Csol. As a result, movements in S6ext are amplified to shifts >7 Å for the Csol in chain A. Conversely, chain D displays a more subtle movement of the S6ext helix (~1 Å), with smaller displacements of ~3 Å in the Csol (fig. S8B). These mismatches in absolute movements further propagate throughout the entire cytosolic shell (Fig. 5, E and F). This likely creates tensions that are released through a disruption of interactions near the Bsol and SPRY2 domains of chains A and D, respectively (Fig. 1C and movie S2).

Notably, in the global refined structure, we observed density for IpCa in two orientations that are related through a twofold symmetry axis (fig. S4A). Both in the local and global refined structures, IpCa pushes one diagonal pair of S6ext helices (chains A and J) further apart than the other pair (chains D and G) (Fig. 5, C and D). From the viewpoint of a single IpCa orientation, the asymmetric break can thus occur in one of two diagonally opposed locations.

Figure S9 compares the different classes obtained for IpCa-bound RyR1 under different conditions. This shows that the degree of asymmetric breaks is substantially less for RyR1 bound to IpCa in the absence of activating ligands (dataset 1). The exact reason for this observation is unclear, but Ca^2+^ is known to bind at the interface between the CTD and Csol. It is therefore part of the pathway that connects movements in the S6ext helix to the Csol and beyond. One possibility is that the lack of Ca^2+^ at this interface reduces local rigidity, allowing for the release of internal tensions.

### Subconductance is the result of an extended permeation pathway

**Fig. 6.**
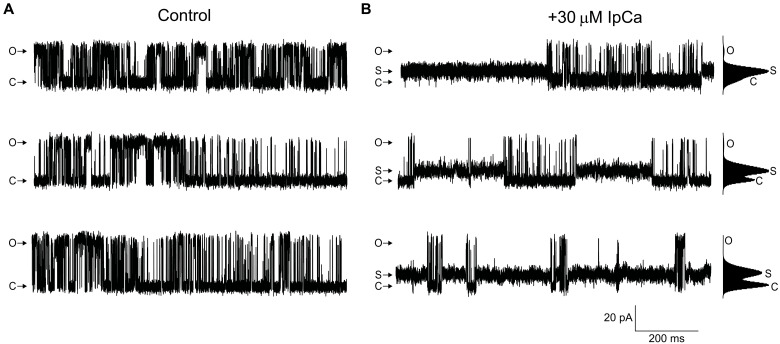
Subconductance caused by IpCa. (**A**) Representative single-channel recordings of rabbit RyR1 reconstituted in planar lipid bilayer from three independent experiments in the presence of 30 μM free Ca^2+^. Recordings were carried out under symmetrical conditions ([*cis*] = [*trans*]) at a holding potential = +60 mV. Openings are shown as upward deflections (C, closed; S, subconductance; O, open). (**B**) The same channels after addition of 30 μM IpCa to the *cis* chamber, showing segments containing all three states. The open state is very rare at the high concentration of IpCa used, as indicated by the current histograms, obtained from 2-min segments of channel activity after IpCa addition. C, S, and O mark the closed, subconductance, and open states of the channel, respectively.

IpCa is known to cause long-lived subconductance states at sub-micromolar concentrations. In the cryo-EM condition, we used high (~30 μM) concentrations of IpCa to saturate the particles. A control experiment using planar lipid-bilayer electrophysiology shows that this concentration still leads to subconductance as observed before (Fig. 6).

**Fig. 7.**
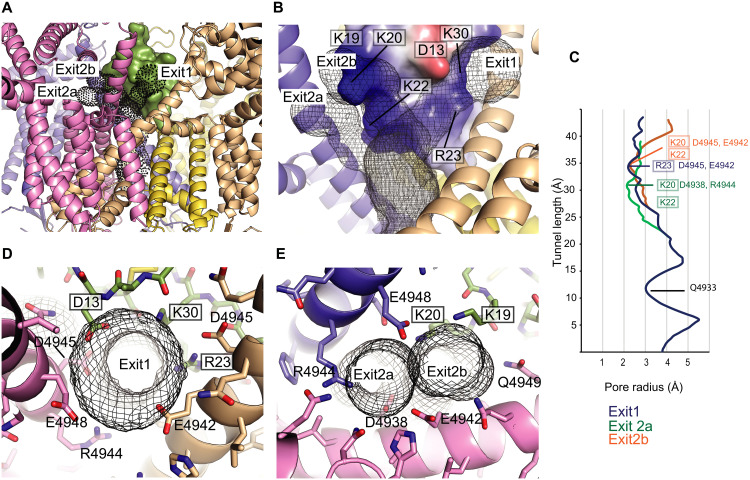
Potential Ca^2+^ permeation pathways through IpCa-bound RyR1. (**A**) Permeation pathways (black dots) determined via *CAVER* (*21*). Shown is a close-up of the transmembrane and adjacent cytosolic region, with different RyR1subunits in cartoon and IpCa in green surface. *CAVER* predicts the main exits in between two different pairs of S6ext helices, one of which bifurcates in two separate options. (**B**) More detailed view of (A), showing the electrostatic potential map of IpCa (blue, positive charge; red, negative charge) with select labels for charged IpCa residues that line the permeation pathways determined by *CAVER* (black mesh). One subunit in “front” has been removed to better visualize the tunnels. (**C**) Tunnel radius relative to the length of the tunnel for the permeation paths shown in (A) and (B). Residues in IpCa (boxed) and in RyR1 (not boxed) near the narrowest part for each pathway are indicated, as well as RyR1 residue Q4933 in the permeation pathway. The starting point for the tunnel calculation is at residue 4894 near the luminal exit. Exit 2a diverges from exit 1 at Gly^4934^ (chain G) and Phe^4940^ (chain J). Exit 2b diverges at Arg^4944^ (chain G) and Asp^4938^ (chain A). (**D**) Detail of the narrowest part of exit 1, which is lined by residues from IpCa (green) and two subunits of RyR1 (pink and wheat). The black mesh delineates the pathway determined by CAVER. Boxed labels are for IpCa residues; unboxed labels indicate RyR1 residues. (**E**) Detail of the narrowest parts of exits 2a and 2b, which is lined by IpCa (green) and two RyR1 subunit (pink and blue).

Because IpCa is located on the fourfold symmetry axis, permeating Ca^2+^ ions need to follow a path away from the central axis. In an open RyR1 without calcin bound, Ca^2+^ can also exit through four large symmetry-related gaps in between the S6ext helices of neighboring subunits. However, IpCa completely closes off one of these gaps, with Thr^26^ forming a plug that prevents any exit (fig. S10). Tunnel calculation using CAVER (*21*) shows two major, and one possible minor, exit pathways through the remaining gaps (Fig. 7 and fig. S10). The one with the largest minimum radius (“exit 1”) is lined by IpCa residues Asp^13^, Arg^23^, and Lys^30^, and RyR1 residues Asp^4938^, Glu^4942^, Arg^4944^, Asp^4945^, and Glu^4948^, located within S6ext of different subunits (Fig. 7), with a minimum radius of ~2.3 Å. Although subconductance may be the result of slightly smaller radii, there are several positively charged residues in IpCa that line the extended permeation pathway, which would result in an electrostatic barrier and thus reduced permeation. An alternative pathway further bifurcates and has minimum pore radii of ~2.2 Å for both (exit 2a and exit 2b). In both cases, this narrowest part of the tunnel is lined by IpCa residues Lys^20^ and Lys^22^, and the electrostatic surface potential is even more positive than for exit 1 (Fig. 7B).

A third tunnel with a smaller radius (~1.8 Å) can be found at the lateral opening near Arg^24^ (“exit 3,” fig. S10D). The narrower radius, along with the presence of three positively charged residues at the constriction point, likely provides a sizeable energetic barrier. Thus, on the basis of minimum radius and electrostatic surface potential, exit 1 may form the most likely exit pathway for Ca^2+^ ions. In vejocalcin and intrepicalcin, two calcins with the highest degree of subconductance (*2*, *14*), Lys^30^ is replaced with a neutral Gln and Lys^22^ is replaced by Ser (fig. S5), which likely lowers the energetic barriers through multiple exits, explaining the higher subconductance. In urocalcin, which also displays a high subconductance (55%), Lys^20^ is replaced by Ser, which would substantially lower the energetic barrier through exits 2a and 2b. Thus, the proposed pathways seem to be in general agreement with the degree of subconductance reported for various calcins (*2*, *14*).

### PKA neutralizes IpCa through direct steric hindrance at Thr^26^

**Fig. 8.**
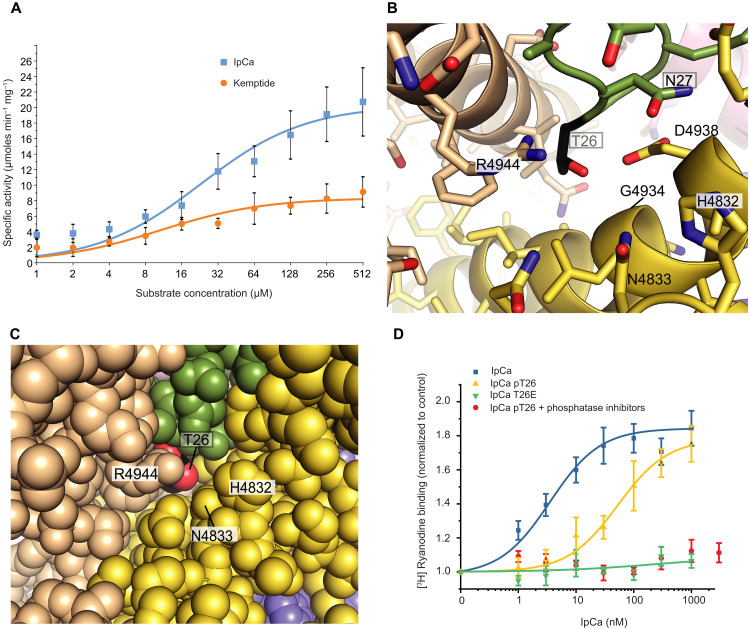
Phosphorylation of IpCa Thr^26^. (**A**) Michaelis-Menten plot for PKA in the presence of IpCa (blue) or kemptide control peptide (orange), showing that IpCa is a good substrate (see table S2 for enzymatic parameters) (*n* = 6; technical replicates). (**B**) Detail around IpCa residue Thr^26^. IpCa is colored in green with Thr^26^ in black. RyR1 subunits are shown in beige and yellow, using the same color scheme as in Fig. 1. (**C**) View of Thr^26^, further zoomed out, with all atoms represented as van der Waals spheres. The colors are as in (B). Calculations using Areaimol (CCP4) indicate that the residue is mostly buried with only 4.9-Å^2^ solvent-accessible surface area (corresponding to 3% of the accessibility of Thr in a G-T-G peptide). (**D**) [^3^H] Ryanodine binding curves to skeletal muscle heavy SR vesicles in the presence of IpCa-WT (blue line and squares), IpCa-pT26 (yellow line and triangles), and IpCa-T26E (green line and upside-down triangles). Median effective concentration values for IpCa-WT and for IpCa-pT26 were 6 ± 2 nM and 42 ± 10 nM, respectively (mean ± SEM for *n* = 3 separate experiments). Red circles represent SR vesicles in the presence of IpCa-pT26 and phosphatase inhibitors.

As calcins are able to permeate the plasma membrane, they are subject to potential posttranslational modifications by the host cell. The 22–30 loop in IpCa contains a consensus motif (RRXS/T) for PKA phosphorylation at residue Thr^26^. This motif is conserved in all calcins except opicalcin-2 (fig. S5). A direct superposition of IpCa with a crystal structure of PKA bound to substrate (*22*) shows that IpCa cannot fit in the pocket without substantial structural changes (fig. S11). We performed phosphorylation enzyme kinetics to determine the degree of phosphorylation by PKA, which shows that PKA has a higher *k*_cat_ and *V*_max_ (but a less favorable *K*_M_) toward IpCa compared to kemptide, a commonly used reference peptide for PKA enzymatic assays (table S2 and Fig. 8A). This indicates that IpCa, and likely most calcins, are excellent PKA substrates (*11*) and highlights the intrinsic flexibility of the 22–30 loop.

What would be the structural and functional impact of IpCa phosphorylation by PKA? Thr^26^ has a minimal solvent accessibility in the complex and forms extensive interactions with RyR1 (Fig. 8, B and C). Addition of a phosphate group would lead to substantial clashes and thus preclude binding. To verify this, we prepared two variants of IpCa, containing either a phosphomimetic (T26E), or a phosphorylated Thr^26^ (pT26). Collection of cryo-EM data on RyR1 in the presence of either variant failed to show any density for IpCa (fig. S3), and [^3^H] ryanodine binding assays show that IpCa-T26E was completely inert (Fig. 8D), in agreement with the steric hindrance model. However, IpCa-pT26 still showed an increase in [^3^H] ryanodine binding, albeit with a sevenfold reduced potency (Fig. 8D). We reasoned that this might be due to the presence of contaminating phosphatases in the SR vesicle preps, which could dephosphorylate a substantial fraction of IpCa-pT26. When the latter was tested in the presence of phosphatase inhibitors, it was no longer able to stimulate [^3^H] ryanodine binding (Fig. 8D). Thus, the combined cryo-EM and functional assays show that phosphorylation of IpCa by PKA effectively neutralizes the calcin, and in a cellular milieu, its potency would depend on the relative activity of PKA and phosphatases. We also note that this confirms the general presence of phosphatases in SR vesicle homogenates, which may partly explain the often confusing and contradictory findings on functional studies of RyRs derived from SR vesicles, instead of full purification. The lack of IpCa-pT26 effect on RyR1 due to the steric hindrance imposed by the phosphate group argues against a study on maurocalcin proposing that phosphorylated calcins can bind to and antagonize RyR1 activity (*11*). Despite prolonged incubation (>1 hour), we did not observe any density for IpCa-pT26 near RyR1.

## DISCUSSION

Calcins are cell-penetrating peptides that cause stable subconducting states via a dual effect. Their binding prevents a closed conformation of the channel pore through direct steric hindrance with the extensions of the inner helices of the channel pore, and they generate constrictions within the cytosolic assembly that limit the flow of Ca^2+^ ions. These constrictions are lined by positively charged calcin residues and directly explain the stable subconductance that has been measured for calcins. Our analysis shows that there are multiple possible pathways through which Ca^2+^ ions could exit the IpCa-bound RyR, and these differ substantially in terms of radii and electrostatic profiles. One pathway, lined by IpCa residues Arg^23^, Asp^13^, and Lys^30^, forms the most likely exit, as it forms the widest tunnel and is the only one that contains a negatively charged residue in IpCa. However, molecular dynamics simulations will prove highly useful for determining the relative contribution of each of these alternatives and to compare the permeations of different cations.

Our structural and functional data directly oppose a previously proposed binding site for calcins in the binding region for CaM (*16*). This previous study was performed using biotinylated IpCa, and in complex with streptavidin, which may have interfered with the binding site observed in our study. The study was also at much lower resolution, and therefore, any difference density observed could be due to conformational changes that result from binding to another location, similar to previous conflicting results around the N-terminal region of the RyR (*23*). We observed that IpCa can cause substantial conformational changes in the cytosolic shell, including asymmetric breaks close to the CaM binding site. C4 symmetry averaging at lower resolution may thus inadvertently have led to the interpretation that IpCa resides near the CaM binding site. Our observation that CaM and IpCa bind at different locations agrees with previous functional data, which show that IpCa can still enhance [^3^H] ryanodine binding in the presence of 3 μM CaM (*24*).

Subconducting states have also been observed in the presence of low concentrations of ryanodine. At higher concentrations, however, addition of ryanodine results in full block (*25*). Such a dual effect has not been observed for calcins, and single-channel recordings in this study also show that 30 μM IpCa still results in subconductance. A previous cryo-EM study has resolved density for ryanodine in the pore-forming region, inside the inner vestibule below Gln^4933^ (*15*). As this site is >13 Å away from IpCa, ryanodine and IpCa can bind to RyRs simultaneously (fig. S12). Whereas IpCa causes subconductance by creating extra constrictions outside of the transmembrane region, subconductance due to ryanodine binding is likely the result of partial occlusion within the pore itself (*15*). Binding of multiple ryanodine molecules in the pore may underlie the full block observed at higher concentrations, but this remains to be confirmed via high-resolution structures. Superposing our IpCa-bound structure with the previously solved structure in the presence of ryanodine, shows that, similar to IpCa, ryanodine binds to open RyRs. However, the S6ext helices are bent outwards more in the presence of IpCa (fig. S12), and no major asymmetry has been reported in the presence of ryanodine (*15*). Thus, the precise effects of ryanodine and IpCa on RyRs differ.

[Cyclic adenosine monophosphate (AMP) (cAMP)]–dependent PKA is stimulated through the β-adrenergic signaling pathway and affects many intracellular targets, including RyRs (*22*). We find that PKA efficiently phosphorylates IpCa, abolishing its ability to bind RyRs through direct steric hindrance. In a physiological setting, the availability of IpCa will depend on the relative abundance of activated kinases and phosphatases that can affect the phosphorylation status of Thr^26^.

Hundreds of disease-causing mutations in RyRs have been linked to various pathologies, including malignant hyperthermia, stress-induced arrhythmia, and central core disease (*5*). Most cause a gain-of-function phenotype through inducing conformational changes that prime channel opening (*18*, *26*). In addition, aberrant posttranslational modifications of RyRs that enhance their channel opening have been suggested to contribute to a range of other pathologies including Alzheimer’s disease, atrial fibrillation, and heart failure (*27*–*30*). Thus, molecules that can selectively block RyRs may have therapeutic benefits.

Protein-based therapeutics have the advantage over small molecules that they can be more selective, by virtue of larger binding interfaces. They are mostly limited to extracellular binding sites, but calcins offer a unique template thanks to their ability to cross membranes. As the IpCa-bound structure reveals constriction points for a permeating Ca^2+^, lined in part by IpCa itself, modifications in the calcin sequence toward bulkier residues could result in full block. The binding site for calcins is fully conserved among the RyR isoforms, but additional targeting strategies toward specific tissues could be considered (*31*). Previously, it was also found that IpCa has little effect on RyR1 in skeletal muscle myotubes, suggesting that its coupling to Ca_V_1.1 prevents the binding or modulation by IpCa (*32*). Thus, therapeutic strategies involving calcin homologs may mostly focus on cardiac applications. Although most calcins are likely neutralized by PKA, the observation that opicalcin-2, which lacks Thr^26^, can still bind with high potency shows the potential to create calcin analogs that are insensitive to kinase activity.

## MATERIALS AND METHODS

### Expression and purification of GST-FKBP12.6 and CaM_1234_

Human FKBP12.6 was cloned into a modified pGEX vector with an N-terminal hexahistidine tag, followed by glutathione *S*-transferase (GST) and a cleavage site for tobacco etch virus (TEV) protease. CaM with mutations D20A/D56A/D93A/D129A (CaM_1234_), which is unable to bind calcium ions (*19*), was cloned into a modified pET-28 vector containing an N-terminal hexahistidine tag followed by maltose binding protein and a TEV protease cleavage site. Flasks containing autoinduction media (*33*) were inoculated with colonies from freshly made plates of transformed *Escherichia coli* Rosetta (DE3) pLysI strain (Novagen) for both proteins. Cells were grown at 37°C with shaking (200 rpm) for 6 hours before changing temperature to 24°C and grown for additional 24 hours before harvesting. Cells were harvested by centrifugation at 8000*g* for 15 min at 4°C. Cells expressing FKBP12.6 were resuspended and lysed via sonication in lysis buffer [10 mM Hepes, 250 mM KCl, deoxyribonuclease I (DNase I; 25 μg/ml), lysozyme (25 μg/ml), 10 mM imidazole, 5 mM MgCl_2_, and 1 mM phenylmethylsulfonyl fluoride (PMSF), with final pH adjusted to 7.4]. The lysate was centrifuged at 40,000*g* for 30 min at 4°C, and the supernatant was filtered through a 0.45-μm filter. The sample was loaded onto 10 ml of pre-equilibrated immobilized metal affinity column (HisTRAP MC, Cytiva), washed with 10 column volumes (CV) of buffer A [10 mM Hepes and 250 mM KCl (pH 7.4)] containing 10 mM imidazole, and eluted with 5-CV buffer A supplemented with 500 mM imidazole (final pH 7.4). After dialyzing 3 to 4 hours against a buffer containing 10 mM tris–HCl (pH 8.8) and 20 mM KCl, small amounts of GST-FKBP12.6 precipitate were removed by centrifugation, and the proteins were further purified by an anion exchange column (HQ, Cytiva) using buffer C [10 mM KCl; 10 mM tris-HCl (pH 8.8)] and buffer D [1 M KCl; 10 mM tris-HCl (pH 8.8)], with a linear gradient of 0 to 55% buffer D. The FKBP12.6- and CaM_1234_-containing fractions were concentrated and injected on a gel-filtration column (Superdex 75, Cytiva) and eluted with buffer A. Peak fractions were collected, concentrated, supplemented with 20% glycerol, flash-frozen with liquid nitrogen, and stored at −80°C.

### Expression and purification of mouse cAMP PKA for kinase assays

The catalytic subunit of mouse cAMP PKA (α isoform) referred to as PKAc, were expressed in *E. coli* Rosetta (DE3) pLysI competent cells using autoinducing media (*33*). Flasks were inoculated with several colonies from freshly transformed plates. After initial incubation at 37°C for ~8 hours, the flasks were incubated at 18°C for 40 to 48 hours with shaking (250 rpm). Six to eight hours before harvesting, isopropyl-β-d-thiogalactopyranoside was added to a final concentration of 0.4 mM to further boost expression. Cells were lysed via sonication in 10 mM Hepes (pH 7.5) and 250 mM KCl, 5 mM MgCl_2_, and 10 mM Imidazole supplemented with DNase I (25 mg/ml), lysozyme (25 mg/ml), and 0.1 mM PMSF, and 0.5 to 1 mM tris(2-carboxyethyl)phosphine hydrochloride (TCEP). The lysate was either applied to a HisTrap column (Cytiva), washed with 5 CV of buffer A, and subsequently eluted with buffer A plus 500 mM imidazole (pH 7.5). Elution was then run on a prepacked amylose column (New England Biolabs) using buffer A supplemented with 10 mM β-mercaptoethanol (βME) for equilibration and wash. Samples were eluted using buffer A plus 10 mM maltose and 10 mM βME. Eluent was dialyzed overnight against buffer A plus 10 mM βME and was cleaved simultaneously with recombinant TEV protease. The samples were then run on a Poros MC (Tosoh Biosep) column using buffer A, where the flow-through was collected, and subsequently dialyzed against 10 mM KCl, 10 mM βME, and 20 mM MES (pH 6.5). The protein was applied to a high-performance sulphopropyl column (Cytiva) equilibrated with 20 mM MES (pH 6.5), 10 mM KCl, and 10 mM βME. Elution fractions were collected with a gradient of 0 to 23% of buffer containing an additional 1 M KCl over 23 CV.

### Kinase assay

PKAc was concentrated to 500 μM, aliquoted, and stored in kinase buffer containing 20 mM Hepes (pH 7.4), 150 mM KCl, 4 mM TCEP, 5 mM MgCl_2_, and 30% glycerol and stored at −70°C in 0.5-ml tubes prior for use in kinase assays. All ADP-Glo Max reagents (Promega) were thawed and brought to room temperature before use. Each 0.5-ml PKAc tube was thawed and diluted to 0.5 μM in the kinase buffer without the glycerol. Kemptide and IpCa substrates were diluted to 1 mM in kinase buffer (without glycerol) from 10 mM stock made from lyophilized powder. Using a twofold dilution series, substrate concentrations ranged from 512 to 0.5 μM. In addition, a reaction with no substrate was added for a total of 12 reactions using a 20-μl reaction volume. One microliter of ATP (from the ADP-Glo Max kit) was used to reach a final concentration of 1 mM ATP in the reaction volume. Reaction was started by adding 1 μl of PKAc to reach 25 nM for each reaction. All reactions were carried out at room temperature for 30 to 60 min. Duplicates of each reaction tube were plated on a Corning 384-well solid white polystyrene microplates (Thermo Fisher Scientific) and used for downstream detection and luminescence readings. The average values of each duplicate reaction were used as a single replicate. Thus, for the total of six replicates, 12 reactions were used. Readings are based on relative luminescence units, which are converted into moles of ATP hydrolyzed using an adenosine diphosphate–ATP standard curve according to manufacturer’s protocol (Promega). Each standard curve was run in duplicate where the average was used to calculate moles of ATP hydrolyzed in each kinase reaction. Luminescence readings were recorded from the microplate wells using a PerkinElmer Victor X4.

### Synthesis and purification of IpCa

IpCa, IpCa-T26E, and IpCa-pT26 were synthesized by the solid-phase methodology as described previously (*2*, *34*). In brief, linear peptides were synthesized on NovaSyn TGA resin (Novabiochem) with Fmoc–amino acids. After cleavage with 90% trifluoroacetic acid (TFA) for 4 hours at room temperature, the crude linear peptides were extracted with 20% acetic acid and lyophilized. The cyclization reaction to make the corresponding disulfide bridges of the molecule was conducted in the folding buffer [20 mM Na_2_HPO_4_, 0.1 M NaCl, 5 mM glutathione (reduced form), and 0.5 mM oxidized glutathione]. After pH adjustment to 7.9 with 1 M NaOH, the reaction mixture was allowed to oxidize at room temperature for 4 hours. After oxidation of the disulfide bonds, the peptide solution was acidified with formic acid to pH 3.0 and purified in a C18 analytical column (218TP54 Vydac) run with linear gradient from solvent A (0.12% TFA in water) to 60% solvent B (0.1% TFA in acetonitrile) over 60 min. The resulting peptides were analyzed by mass spectrometry and amino acid sequencing.

### Purification of intact RyR1 from rabbit skeletal muscle

WT rabbit skeletal muscle was obtained from Pel-Freeze Biologicals. Approximately 200 g of frozen rabbit skeletal muscle was blended for 120 s in 2 × 500 ml of 10 mM tris/maleate (pH 6.8), 1 mM dithiothreitol (DTT), 1 mM EDTA, 200 μM PMSF, and 1 mM benzamidine. The mixture was centrifuged at 4°C for 10 min at 7000*g*. The supernatant was filtered through a cheesecloth and centrifuged at 4°C for 40 min at 40,000*g*. Pellets were solubilized in buffer S1 containing 20 mM Hepes (pH 7.4), 1 M KCl, 2 mM TCEP, 150 μM PMSF, 1 mM EGTA, 1% CHAPS, and 0.2% soybean phosphatidylcholine with 100 μl of protease inhibitor cocktail (Protease Inhibitor Cocktail Set III, EDTA-Free, Calbiochem). After stirring for 1 hour at 4°C, the solubilized membranes were diluted with 120 ml of buffer S2 (as for buffer S1 but lacking 1 M KCl). His-GST-FKBP12.6 (~5 mg) was then added to the solubilized membranes, with incubation for 2 hours at 4°C. After ultracentrifugation for 45 min at 200,000*g*, the supernatant was filtered and mixed with 2 to 3 ml of GS4B resin (Cytiva) pre-equilibrated with buffer S3 containing 20 mM Hepes (pH 7.5), 0.5 M KCl, 0.5% CHAPS, 0.2% soybean phosphatidylcholine, 1 mM EGTA, 2 mM TCEP, 150 μM PMSF, and 100 μl of protease inhibitor cocktail, with stirring for 3 hours at 4°C. The mixture was poured into a column and washed with 10-CV buffer S3. Next, 10 ml of buffer S4 (identical to S3 except with 75 mM Hepes) together with 1.5 mg of TEV protease was added to the column and incubated overnight to elute RyR1 from the column. The eluents were concentrated to 500 μl, applied to a gel-filtration column (Superose 6 10/300 GL, Cytiva) and eluted with buffer S5 containing 25 mM Hepes (pH 7.5), 0.25 M KCl, 0.375% CHAPS, 0.001% 18:1 (Δ9-*cis*) phosphatidylcholine (DOPC), 200 μM PMSF, 200 μl of protease inhibitor cocktail, and 2 mM EGTA (dataset 1) or 30 μM free Ca^2+^ buffered with 2 mM EGTA (datasets 2 to 5). Peak fractions containing RyR1 complexes were combined and concentrated to ~10 to 16 mg/ml. Concentration was estimated using NanoDrop [absorbance at 280 nm, 1% (w/v) = 1.0] and used same day for plunging.

### Electron microscopy

For activating conditions (datasets 3 to 5), RyR1 samples were diluted to contain 30 μM buffered free Ca^2+^, 80 μM CaM_1234_, 5 mM caffeine, and 8 mM (dataset 3) or 5 mM (datasets 4 and 5) ATP. Purified IpCa (powder) was first diluted to 1 mM in buffer S5 without EGTA or Ca^2+^ and then added to the RyR1 samples to a final concentration of 30 to 80 μM. Samples were incubated in the presence of IpCa (including pT26 and T26E) up to an hour before plunging. RyR1 particles from each condition were assessed by negative-stain EM. Four microliters of purified RyR1 diluted to 0.01 to 0.05 mg/ml was applied to glow-discharged 400-mesh copper grids (Electron Microscopy Sciences) and stained with 0.7% (w/v %) uranyl formate. Negatively stained EM grids were imaged on a Tecnai G2 Spirit microscope (Thermo Fisher Scientific) operated at 120 kV or a Talos with a Ceta camera operated at 120 kV.

For cryo-EM, RyR1 samples with a final concentration of 10 to 12 mg/ml were used. The samples were applied to holey carbon R2/2 grids (Quantifoil). Grids were blotted for 3 s with −5 to −15 blot force applied and subsequently flash-frozen with liquid ethane using a Vitrobot IV (Thermo Fisher Scientific). Grids were transferred to a Titan Krios G2 electron microscope (Thermo Fisher Scientific) operating at 300 kV. A total of 596 movies for the EGTA (dataset 1), 1059 movies for 30 μM free Ca^2+^ (dataset 2), and 7277 movies for activating condition in the presence of wild-type IpCa (dataset 3) were collected using the automated collection system EPU (Thermo Fisher Scientific) at a nominal magnification of ×75,000 in counting mode on a Falcon III detector calibrated to pixel size of 1.09 Å respectively using a gold cross grating. Total dose was 50 e−/Å2 applied equally over 60s and 48 frames.

A total of 6972 and 16,811 movies for activating conditions in the presence of phosphorylated pT26-IpCa (dataset 4) and phosphomimetic T26E-IpCa (dataset 5), respectively, were collected on a Titan Krios equipped with a Selectris energy filter (Thermo Fisher Scientific) and a Falcon IV detector (Thermo Fisher Scientific) calibrated to pixel size of 0.98 Å using a gold cross grating. Automated data collection was done with the EPU software package. Movies for dataset 4 were pooled from two separate collections, one with 2282 and the other with 1505 exposure fractions collected at a nominal magnification of ×130,000, with a dose rate of 5.7 e^−^/pix per second and 7.65 e^−^/pix per second respectively. Movies for dataset 5 were pooled from two separate collections, one with 2711 and the other with 14,100 exposure fractions collected at a nominal magnification of ×130,000. Defocus for all datasets ranged from −1 to −2.5 μm, with a total dose of 50 e^−^/Å^2^.

For datasets 3 to 5, the pixel size was further calibrated by real-space correlation with a crystal structure of the N-terminal 3 domains of the receptor [Protein Data Bank (PDB) ID: 2XOA] after obtaining the initial reconstruction. A maximum was observed at 1.07 Å for dataset 3, while a maximum of 0.94 Å was obtained for datasets 4 and 5. Motion correction was redone after calibration of pixel size for final map reconstructions.

### CryoEM data processing

A detailed schematic of the image-processing pipeline for each dataset is presented in figs. S1 to S3. All movies were imported and processed using cryoSPARC (v3.2) (*35*). EER formatted movies for datasets 4 and 5 were up sampled by 2 from 0.94- to 0.47-Å pixel size during import. Movies were patch motion–corrected and defocus-estimated as implemented in cryoSPARC. Movies were then curated on the basis of ice thickness, contrast transfer function (CTF) resolution estimation (only images with CTFs <7 Å were kept), motion curve, and estimated astigmatism. Two separate particle picking pipelines were used and combined for final particle stack (figs. S1 to S3). Two-dimensional (2D) templates were generated from a dataset using 2D classification of blob-picked particles (datasets 1 to 3, and 5) or templates with different orientation were generated from a previous RyR1 3D reconstruction [EMD-30067 (*36*)] for dataset 4. Template-picked particles were extracted with a box size of 448 for pixel sizes 1.09/1.07 Å for datasets 1 to 3 or with a box size of 1024, cropped to 512 pixels to obtain 0.94 Å pixel size for datasets 4 and 5. For datasets 4 and 5, we used crYOLO (v1.7.6.4) (*37*) in parallel for particle picking. CrYOLO picked particles were imported and extracted in cryoSPARC as above. Both crYOLO and template-picked particle stacks were separately subjected to 3D classification (ab initio reconstruction) with 4 or 5 classes (*k* = 4 to 5) and a class similarity of 0 to separate junk and RyR from picked particle stacks. Volumes were subsequently Fourier cropped to a box size of 112 or 224, and particles were further refined with heterogeneous refinement to redistribute particles in each class. In all cases, we obtained three to four junk classes and one good RyR class. Template and crYOLO picked particle stacks were then pooled and duplicates were removed. For datasets 3 to 5, another round of 3D classification was used to separate poor RyR looking particles. A 2D classification job was in some cases used for cleanup and removal of low-resolution RyR particles. Raw images, distribution of particle orientation, and 2D class averages are shown in fig. S13.

### 3D subclassification and final map reconstructions

Final RyR pooled particle stacks were further classified with three to five classes depending on size of collection using ab initio 3D reconstruction using class similarity 0.8 to 0.9, followed by heterogeneous refinement (figs. S1 to S3). For datasets 3 to 5 where CaM_1234_ was present, we did not observe any 3D classes that indicated subsaturation of CaM, as expected from using concentrations that are several orders of magnitude above the *K*_d_ (*38*). Final particles and volumes underwent nonuniform refinement (C1 symmetry), CTF refinement, and B-factor sharpening as implemented in cryoSPARC (*35*, *39*). As a separate means to improve the resolution of IpCa and the transmembrane region, we also used particle subtraction to subtract the cytosolic and micelle region, followed by focused or local refinement with C1 symmetry. The mask for local refinement contained the transmembrane region, the S6ext helices, and the bound IpCa. This procedure allowed for a larger relative contribution from IpCa, breaking the twofold symmetric orientations for IpCa observed in the global refinement. The C1 masked refinement was conducted with a fine angular search (0.1°). To improve local density features for an additionally observed transmembrane helix, we also used local refinement with C4 symmetry imposed. All data collection statistics are shown in table S3. Fourier shell correlation curves and local resolutions for deposited maps are presented in figs. S14 and S15.

### Model building

RyR1 + CaM_1234_/Caffeine/ATP/Ca^2+^/IpCa (class 5) and RyR1 + CaM_1234_/Caffeine/ATP/Ca^2+^/pT26-IpCa (class 2), which reflect open channels bound and unbound to IpCa respectively, were built using Coot (*40*) with deposited PDB 6M2W (*36*) as starting template. The individual lobes of apoCaM [PDB ID 6JI8 (*41*)] were placed by rigid-body fitting and manually adjusted in Coot. Models underwent iterative manual remodeling in Coot using density-modified and sharpened maps [using Phenix (*42*) v.1.2.4459 or CryoSPARC’s own implementation of B-factor sharpening]. Phenix real_space_refine was applied to refine the full structure using final maps. The previously unassigned densities and residues of the alpha-solenoid regions were placed using human RyR1 AlphaFold 2.0 (*43*, *44*) model and fitted using Coot. For the phosphorylation domains (2748 to 2939), best fit was achieved using the AlphaFold template. The final model validation statistics were calculated using Phenix (table S3).

### Model analysis

Animations and morphs between different structures were prepared using ChimeraX (*45*). Local and global superpositions are based on TM regions 4929 to 4956. Atomic distances were measured using Coot and ChimeraX. CAVER v3.0 (*21*) was used to determine different tunnels in the transmembrane region. Using a probe radius of 2 Å, this yields tunnels for exits 1, 2a, and 2b. With a 1.5-Å probe radius, an additional tunnel is found for exit 3. Figure S10 also shows a diagram generated by HOLE (*27*) for the most likely exit pathway (exit 1). FSC curves and kinase assays were plotted using GraphPad Prism 8.0.

### Mutagenesis of mouse RyR2 and rabbit RyR1

The mutations of RyR2 residues that binding to IpCa (D4867A, G4870R, D4874A, E4877A/Q4878A, and E4881A/D4882A) were made in a partial construct containing the C-terminal 6041 nucleotides of the mouse RyR2 cDNA (pcDNA3-RyR2-Cassette2), which was flanked by two unique restriction sites (Bsi WI and Not I). Site-directed mutagenesis was performed using the QuikChange Lightning mutagenesis kit (Agilent) following the manufacturer’s recommendations. The partial construct was screened for the desired mutations after mutagenesis, and the positive clones were amplified in *E. coli* (TOP10 cells, Thermo Fisher Scientific). The complementary DNA (cDNA) with the desired mutations were digested with Bsi WI and Not I and ligated into the full RyR2 construct (pcDNA5-mRyR2), which was suitable for protein expression in mammalian cells.

The mutations of RyR1 residues that bind to IpCa (D4938A, G4941R, D4945A, E4948A/Q4849A, and E4952A/D4953A) were made in a partial construct containing the C-terminal 2231 nucleotides of the rabbit RyR1 cDNA (pBluescriptII-rabRyR1-Cterm), which was synthesized commercially. Site-directed mutagenesis was performed using the QuikChange Lightening mutagenesis kit (Agilent) following the manufacturer’s recommendations for mutations D4945A, E4948A/Q4849A, and E4952A/D4953A. Mutagenesis for D4938A and G4941R were made by synthesizing a double-stranded DNA flanked by two Msc I restriction enzyme sites and ligated into the pBluescriptII-rabRyR1-Cterm construct. The pBluescriptII-rabRyR1-Cterm construct was screened for each desired mutation, and the fragments flanked by Sac II and Xba I were ligated into another partial construct, which contains the C-terminal 6788 nucleosides of the rabbit RyR1 flanked by Bsi WI and Xba I (pCIneo-rabRyR1-Cassette2). The cDNA with the desired mutations were further digested with Bsi WI and Xba I and ligated into the full rabbit RyR1 construct (pcIneo-rabRyR1), which was suitable for protein expression in mammalian cells.

### Transient expression of mouse RyR2 and rabbit RyR1 in mammalian cells

Human embryonic kidney–293 cells were maintained in Dulbecco’s modified Eagle’s medium supplemented with 10% fetal bovine serum, 1X nonessential amino acids (Gibco, Thermo Fisher Scientific), and 2 mM GlutaMax (Gibco, Thermo Fisher Scientific). The cells were plated on 100-mm tissue culture dishes at 50 to 60% confluency at least 24 hours before transfection. The cells were transfected with a mammalian expressible vector (pcDNA5 or pCIneo) containing the mouse RyR2 or rabbit RyR1 cDNA using the XtremeDNA HP or XtremeDNA 360 reagent (Roche Diagnostics), with a plasmid-to-reagent ratio of 1:3. The cells were collected 48 hours after transfection and washed twice with phosphate-buffered saline (PBS) before cell pellets were flash-frozen or resuspended in lysis buffer containing 25 mM tris/50 mM Hepes (pH 7.4), 137 mM NaCl, 1% CHAPS, 0.5% soybean phosphatidylcholine, 2.5 mM DTT, protease inhibitors (2 μM leupeptin, 100 μM phenylmethylsulphonyl fluoride, 500 μM benzamidine, and 100 nM aprotinin), and phosphatase inhibitors (50 mM NaF, 1 mM b-glycerophosphate, 5 mM sodium pyrophosphate, and 1 mM sodium orthovanadate). The cell suspensions were sonicated on ice and incubated on a rotator at 4°C for 1 hour. The lysates were obtained by centrifuging at 10,000 rpm at 4°C for 10 min to remove insoluble material. Protein concentrations were determined using the Bradford method (Bio-Rad).

### Ryanodine binding assays

[^3^H] ryanodine binding to cell lysates was carried out as previously described (*46*). Briefly, a binding mixture of 100 μl containing 50 μg of RyR2 or 100 μg of RyR1 of cell lysate in binding buffer containing 200 mM KCl, 20 mM Hepes (pH 7.4), 5 nM [^3^H] ryanodine (56.6 Ci mmol^−1^, Dupont NEN, Wilmington, DE), and CaCl_2_ to set free [Ca^2+^] from pCa 8 to pCa 3. The Ca^2+^/EGTA ratios were calculated using MaxChelator (https://somapp.ucdmc.ucdavis.edu/pharmacology/bers/maxchelator/downloads.htm). For experiments to measure IpCa affinity (dose-response curves), the [^3^H] ryanodine binding assays were performed at fixed [Ca^2+^] = 100 nM (pCa 7) for RyR2 and 10 μM (pCa 5) for RyR1. The samples were incubated for 2 hours at 36°C, then filtered on Whatman GF/B glass filters (Whatman, Clifton, NJ) presoaked with 5% polyethylenimine, and washed twice with 5 ml of distilled water using a Brandel M24-R cell harvester (Gaithersburg, MD). Experiments were done in triplicate, and the nonspecific binding was determined in the presence of 20 μM unlabeled ryanodine and was subtracted from each sample.

#### RyR1 proteoliposome reconstitution and single-channel recordings

RyR1 was reconstituted into proteoliposomes as previously described (*18*). Briefly, a 5:3 mixture of 1,2-dioleoyl-*sn*-glycero-3-phosphoethanolamine (DOPE) and 1,2-dioleoyl-*sn*-glycero-3-phosphocholine (DOPC) (Avanti Polar Lipids) were dried into a thin film, followed by overnight incubation in a vacuum chamber. Dried lipids (1 mg) were solubilized with 400 μl of rabbit RyR1 (0.7 mg/ml) in buffer S5, and the mixture was dialyzed for 48 hours using a 3.5-kDa membrane in 20 mM Hepes-KOH (pH 7.5), 250 mM KCl, and 0.1 mM PMSF. Following dialysis, the samples were aliquoted, flash-frozen in liquid nitrogen, and stored at −80°C for later use.

For all experiments, planar lipid bilayers were formed from a mixture of DOPE:DOPC = 5:3 (mol/mol) dissolved in decane with a final lipid concentration of 15 mg/ml. For single-channel recordings, an integrated chip-based recording setup Orbit mini and EDR2 software (Nanion Technologies) was used. Recordings were obtained in parallel with multielectrode cavity array chips (Ionera Technologies). The cis and trans chambers contained symmetrical solutions of recording buffer [20 mM Hepes-KOH (pH 7.5), 250 mM KCl, and 30 μM free Ca^2+^ buffered with 2 mM EGTA] . The free concentrations for Ca^2+^ were calculated with Ca-EGTA Calculator TS v1.3 (*47*) and verified using a PerfectIon Combination Calcium Electrode unit (Mettler Toledo). To promote fusion to prepared suspended bilayers, 10% glycerol was incorporated into the proteoliposomes. In brief, prepared proteoliposomes were mixed 1:1 in recording buffer supplemented with 20% glycerol. The samples were then freeze-thawed and sonicated five times to incorporate the glycerol into the proteoliposome lumen. RyR1 proteoliposomes were added to the cis chamber. All RyR1 measurements were conducted at 22°C and at a constant voltage of 60 mV. After ~1 min of recording of baseline, IpCa was added to a final concentration of 30 μM in the cis chamber. Recordings were filtered at a final bandwidth of 10,000 Hz. Clampfit (v11.2) was used to analyze current traces and generate figures.

### Statistical analysis

A nonlinear regression Michaelis-Menten kinetics fitting function was used in GraphPad Prism 8.0 to calculate *K*_M_,*V*_max_, and *K*_cat_ values for the PKA-IpCa kinase assays. SEMs are reported for each data point as calculated in Prism. Figures and graphs were generated using PyMOL, ChimeraX, and Prism 8.0 unless otherwise noted. [^3^H] Ryanodine binding data were fitted using nonlinear regression analysis in Origin 2018b software (Origin Lab). Hill’s equation was used to determine *B*_max_ (maximum specific binding) and *K*_d_ (half of the [IpCa] needed to reach *B*_max_). Data are presented as means ± SEM, with number of experiments indicated in figure legends. Statistical significance was determined at *P* < 0.05.
