# Supplementary material for: Cryo-EM analysis of scorpion toxin binding to Ryanodine Receptors reveals subconductance that is abolished by PKA phosphorylation
Source: Sci Adv. 2023 May 24;9(21):eadf4936. doi: 10.1126/sciadv.adf4936 (PMC10208580; doi:10.1126/sciadv.adf4936)
Supplement: Supplementary file 1 — Figs. S1 to S15 Tables S1 to S3 Legends for movies S1 and S2 [file sciadv.adf4936_sm.pdf]

Science Advances
AAAS

Supplementary Materials for

Cryo-EM analysis of scorpion toxin binding to Ryanodine Receptors reveals subconductance that is abolished by PKA phosphorylation

Omid Haji-Ghassemi *et al.*

Corresponding author: Hector H. Valdivia, hvaldivia@wisc.edu; Filip Van Petegem, petegem@mail.ubc.ca

Sci. Adv. **9**, eadf4936 (2023)
DOI: 10.1126/sciadv.adf4936

The PDF file includes:

Figs. S1 to S15
Tables S1 to S3
Legends for movies S1 and S2

Other Supplementary Material for this manuscript includes the following:

Movies S1 and S2

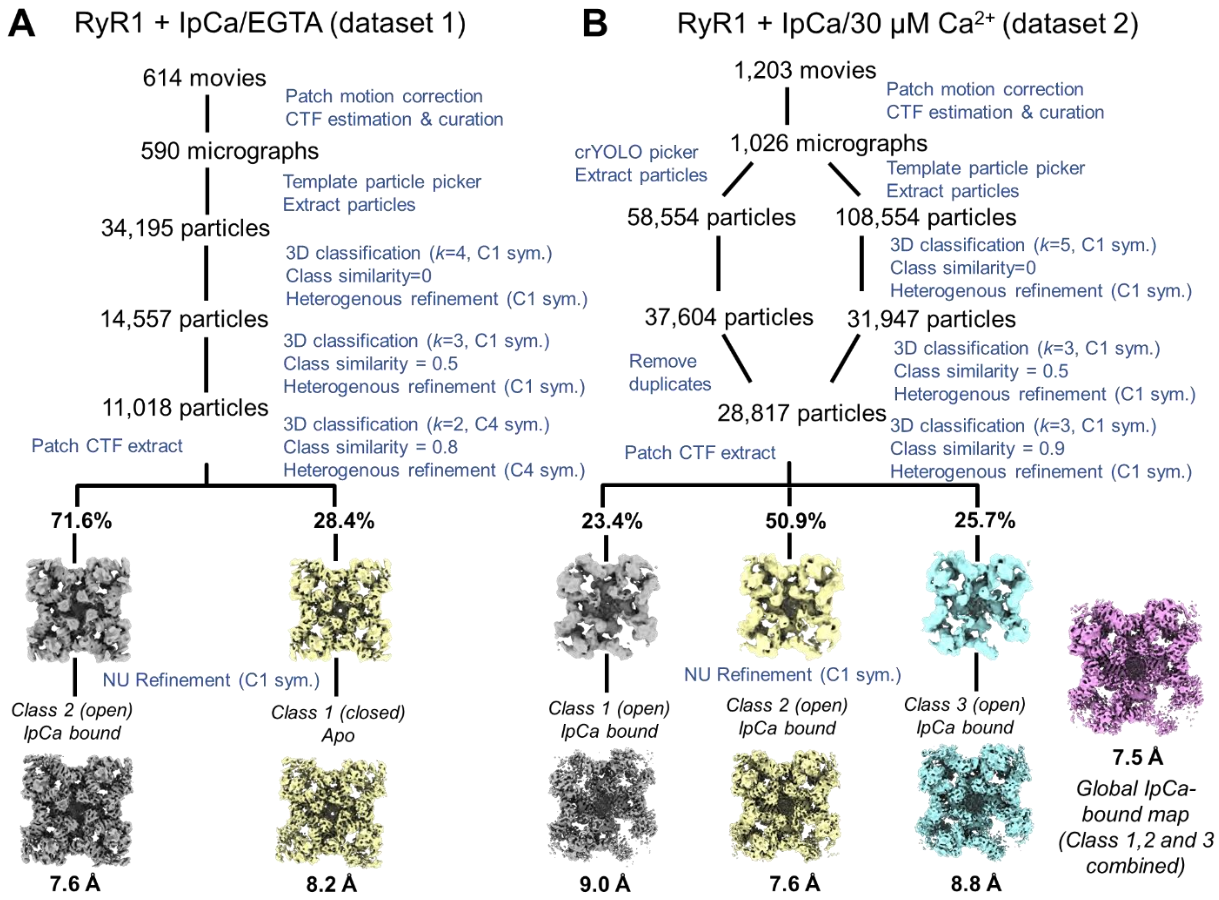

Fig. S1. Cryo-EM processing pipeline for datasets 1 and 2. Cryo-EM data collection, 3D classification and refinement scheme for (**A**) rabbit RyR1 in presence of wild-type IpCa and EGTA and (**B**) rabbit RyR1 in presence of IpCa and 30µM free Ca^{2+}. Note the significant asymmetric breaks visible for the latter dataset.

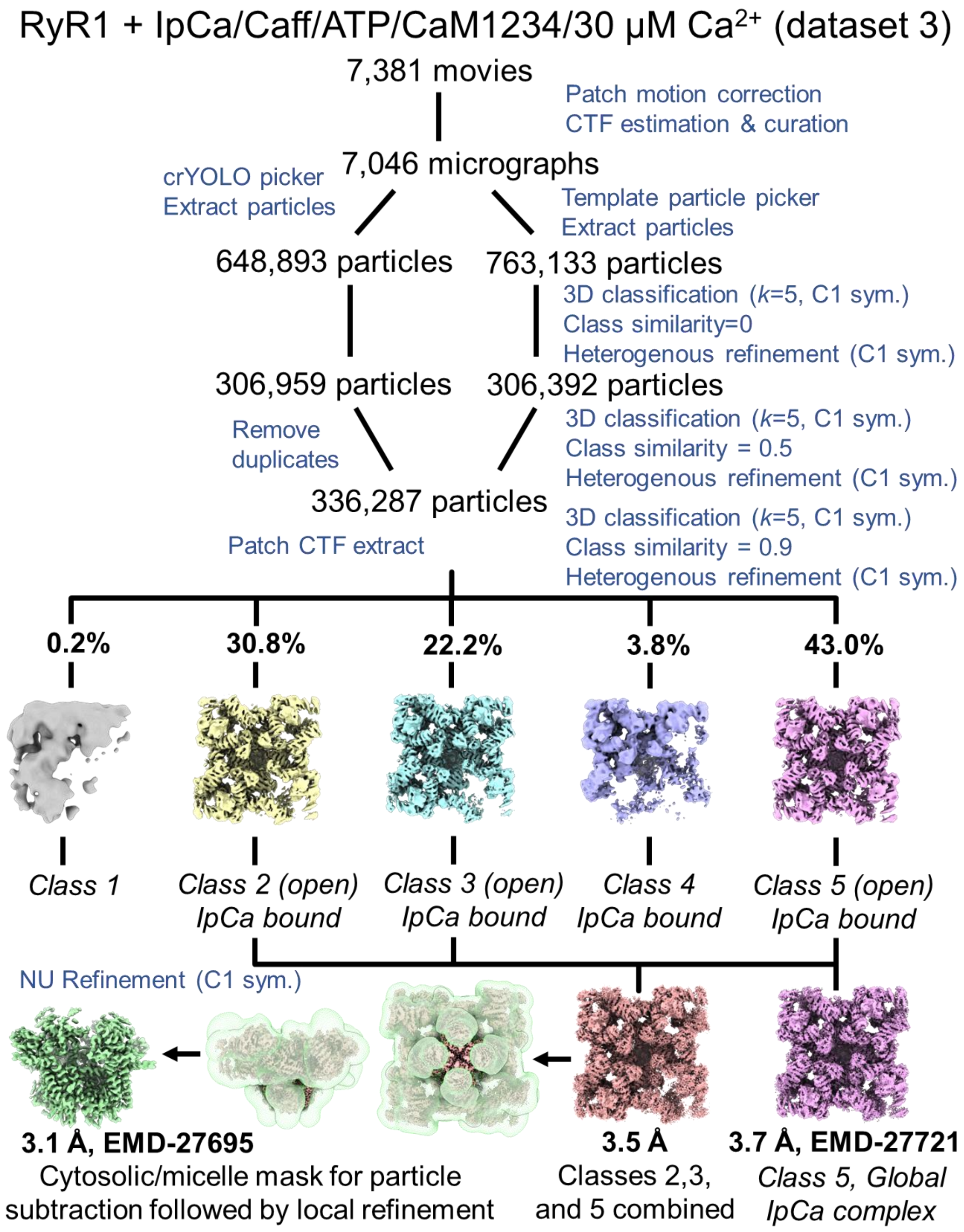

Fig. S2. Cryo-EM processing pipeline for dataset 3. Cryo-EM data collection, 3D classification and refinement scheme for rabbit RyR1 in presence of wild-type IpCa and activating ligands. Note the significant asymmetric breaks in the different classes, most pronounced for Class 3. The class with the least asymmetry (class 5) was chosen for global refinement.

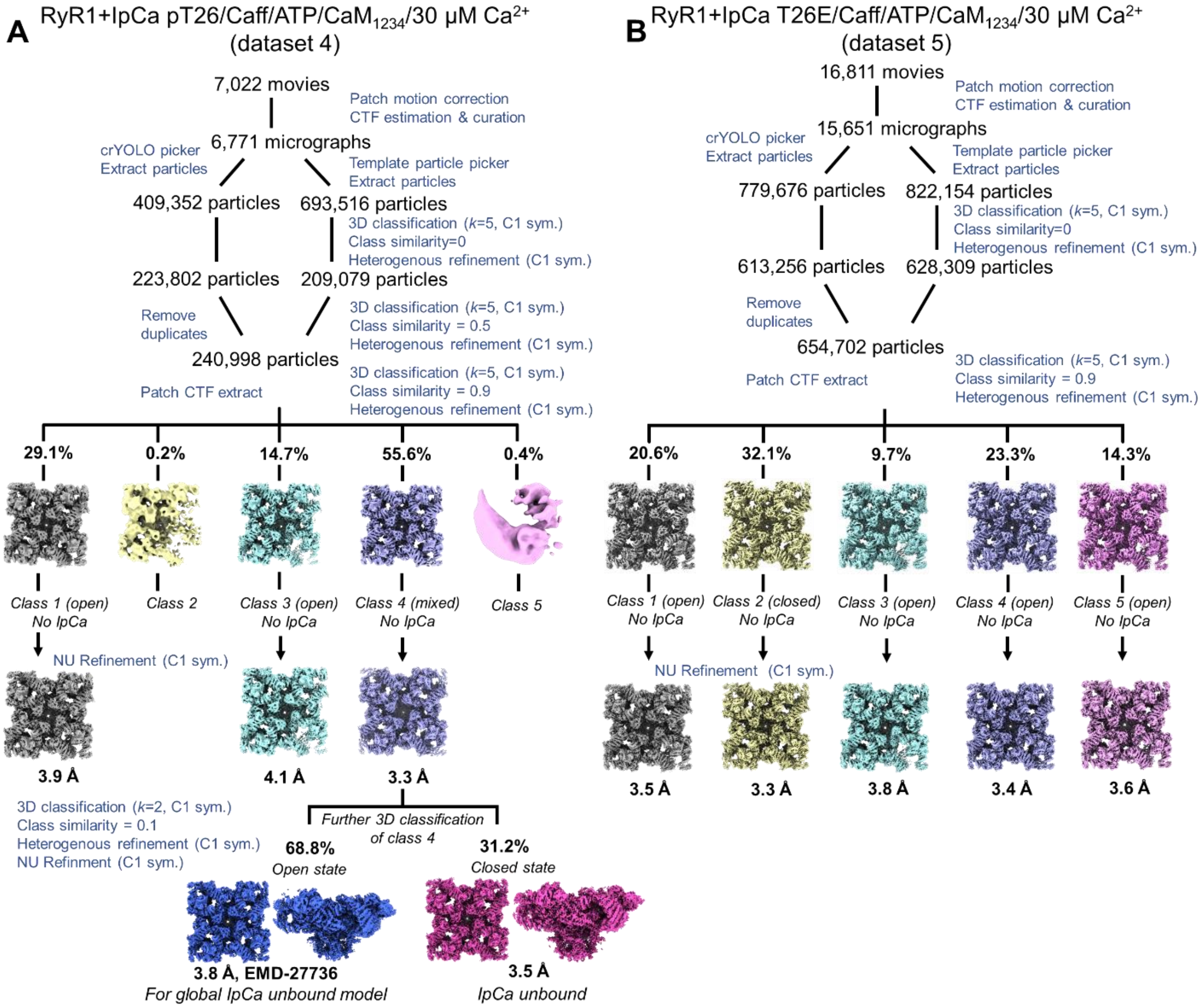

Fig. S3. Cryo-EM processing pipeline for datasets 4 and 5. Cryo-EM data collection, 3D classification and refinement scheme for **(A)** rabbit RyR1 in presence of phosphorylated IpCa (pT26) and activating ligands and **(B)** rabbit RyR1 in presence of IpCa phosphomimetic (T26E) and activating ligands.

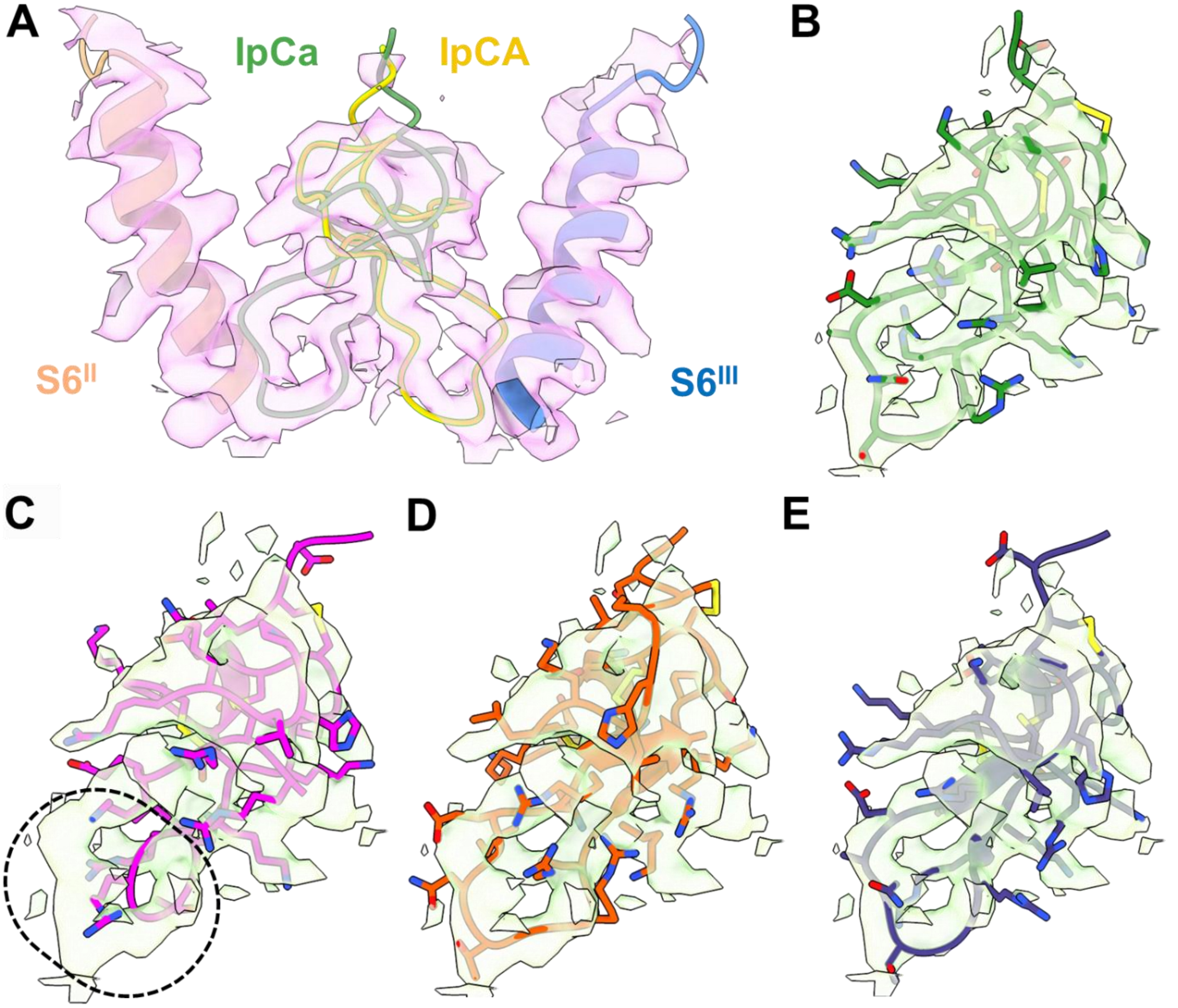

Fig. S4. Density fit for different calcin structures. (A) Globally refined 3.7 Å map (magenta, dataset 3) showing two symmetry-related densities for IpCa that can be superposed by a 180° rotation around a vertical axis. Two IpCa chains are shown in green and yellow. (**B)** Fitted and refined model for IpCa in 3.1Å locally refined map (green). Side chains were modeled based on density features at lower contour levels, and according to their environment in RyR1. (**C**) NMR structure of IpCa (PDB 1IE6) fitted in the same local map as in panel B). The dotted line indicates the 22-30 loop, which does not fit well to the density without readjustment. (**D)** NMR structure of Maurocalcin (PDB 1C6W) fitted to the same local map as in panel b). The 22-30 loop (dotted line) fits the density much better. (**E)** AlphaFold2 model of IpCa fitted to same local map as in panel B). This shows that the IpCa AlphaFold model and the Maurocalcin NMR structure provide better initial fits to the density than the IpCa NMR structure. The only adjustments needed are for side chains, which adopt different conformations when bound to RyR1 (e.g. compare panel E to panel B)

	10 20 30	Subconductance (% from maximum)	Kd,app (nM)
Vejocalcin	ADCLAHLKLC KKNNDCCSKK CSRRGTNPEQ RCR	60%	3.7
Intrepicalcin	ADCLAHLKLC KKNKDCCSKK CSRRGTNPEQ RCR	55%	17.4
Urocalcin	KDCLKKLKLC KENKDCCSKS CKRRGTNIEK RCR	55%**	376
Maurocalcin	GDCLPHLKLC KENKDCCSKK CKRRGTNIEK RCR	48%	26.4
Opicalcin2	GDCLPHLKRC KENNDCCSKK CKRRGANPEK RCR	40%	3.2
Opicalcin1	GDCLPHLKRC KENNDCCSKK CKRRGTNPEK RCR	35%	0.3
Hadrucalcin	SEKDCIKHLQRC RENKDCCSKK CSRRGTNPEK RCR	35%	14.8
Imperacalcin	GDCLPHLKRC KADNDCCGKK CKRRGTNAEK RCR	30%	8.7
Hemicalcin	GDCLPHLKLC KADKDCCSKK CKRRGTNPEK RCR	20%(38%)*	6.9

*38% for hemicalcin reported by Shahbazzadeh et al 2007

**urocalcin was done at a much higher concentration (Xiao et al, 2016)

Fig. S5. Sequence alignment of select calcins isolated from different species. Subconductance and apparent dissociation constant (Kd,app) as reported by Xiao et al (2016) *J Gen Physiol* **147**, 375-94. The subconductance is shown as a % of the maximum conductance obtained when no calcin is present. The sequence numbering on top is for all calcins shown except for hadrucalcin, which contains two extra residues at the N-terminus. Shaded residues are conserved among all calcins.

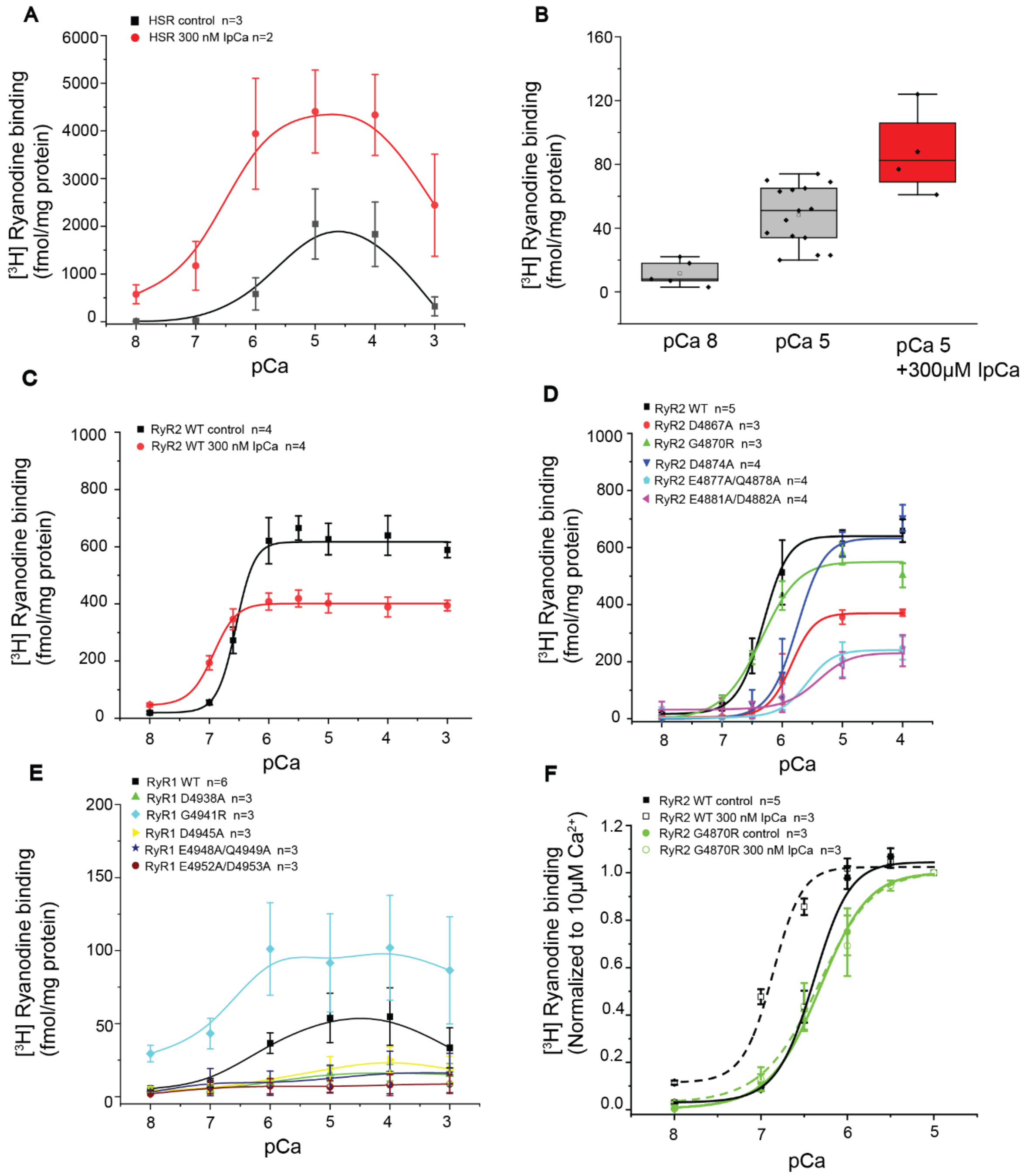

Fig. S6. Ca^{2+} dependence of [^{3}H]ryanodine binding to RyRs and RyR mutants. **(A)** [^{3}H]Ryanodine binding to heavy SR (HSR) vesicles obtained from rabbit skeletal muscle in control and in the presence of 300 nM IpCa (**B**) [^{3}H]Ryanodine binding to vesicles obtained from HEK293-WT RyR1 lysates in three different conditions. Individual data points are shown in box plots that represent the mean±SEM, with whiskers indicating the most extreme data points within the 1.5 interquartile range. **(C)** Ca^{2+} dependence of [^{3}H]ryanodine binding curve from RyR2-WT, obtained from the HEK293 lysates, in the presence and absence of IpCa **(D)** Ca^{2+} dependence of [^{3}H]ryanodine binding curve from WT and mutant RyR2 obtained from HEK293

cell lysates. (**E**) Ca^{2+} dependence of [^{3}H]ryanodine binding curve from WT RyR1 and various mutant RyR1 obtained from HEK293 cell lysates. (**F**) [^{3}H]Ryanodine binding curves from WT and G4870R RyR2 in response to Ca^{2+} in control and in the presence of 300 nM IpCa. The data are normalized to the data obtained at 10 μM Ca^{2+} (WT, black squares and RyR2-G4870R, green circles). RyR2-G4870R abolishes the activating and inhibitory effect that IpCa has on RyR2.

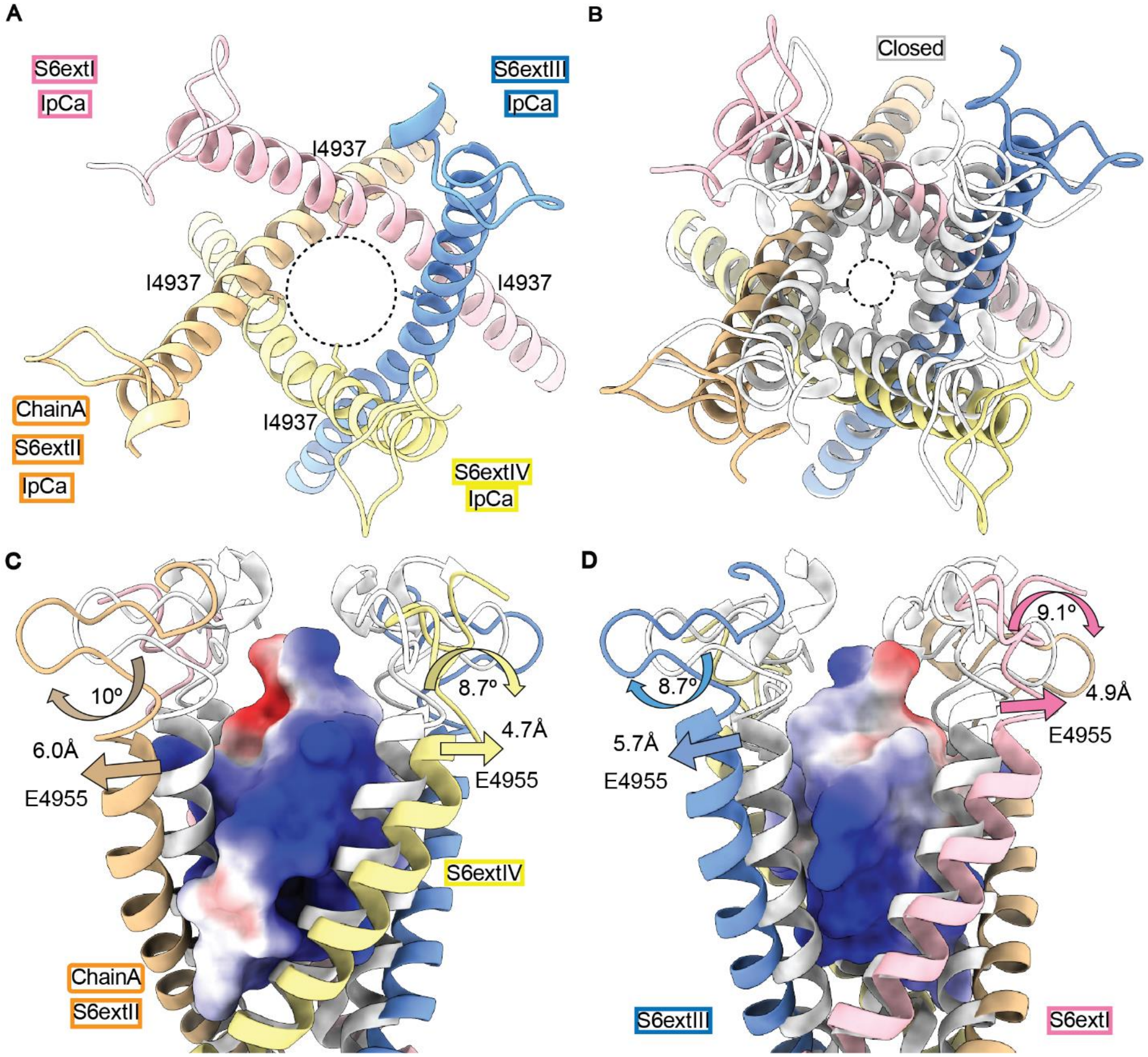

Fig. S7. IpCa clashes with closed RyR1. (**A**) top view of IpCa-bound RyR1 (locally refined structure, dataset 3). The S6/S6ext helices are shown with each subunit in a different color, corresponding to the color scheme used in Figure 1. IpCa is not shown to allow visualization of Ile4937 (labeled), which forms the constriction point in closed channels. The pore is in an open conformation for this structure. (**B**) Same view as in panel A, showing a superposition with closed RyR1 (PDB: 7TZC, white). The side chain for Ile4937 from the closed structure is shown in sticks. (**C**), (**D**) Side views of the same superpositions, with IpCa shown in surface representation and colored according to electrostatic surface potential (blue: positive charge; red: negative charge). The relative movements of the S6 helices are indicated. Without this, there would be major clashes between IpCa and the S6ext helices of every subunit. As S6ext movements also occur during normal opening and closing of RyR1, IpCa likely selects for the open state. However, the possibility remains that very transient states occur where IpCa engages a closed channel.

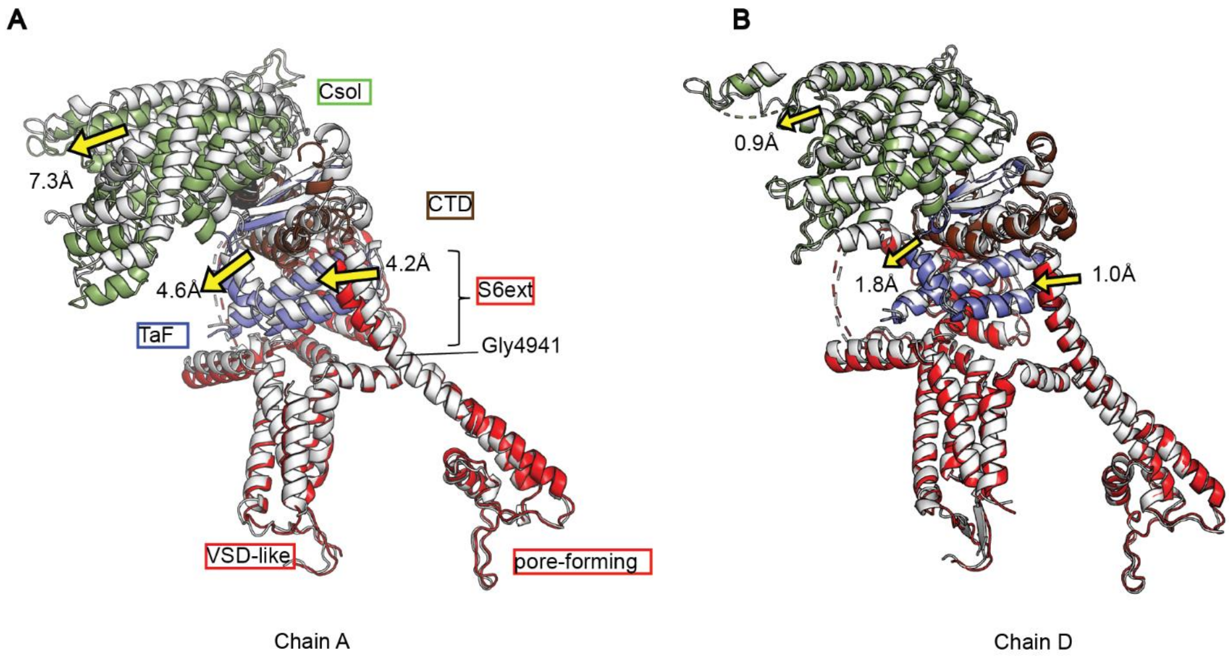

Fig. S8. Asymmetric movements are propagated. (**A**). Superposition of chains A of the global refined structure with IpCa bound (colors, dataset 3) and the reference structure without IpCa bound (white, dataset 4). The superposition is based on the pore forming domain and shows the area encoded by residues 3850-end to highlight how movements are propagated immediately downstream from the S6 extension helix (S6ext). The Transmembrane region and the S6ext are colored in red and various elements are labeled. VSD-like: voltage-sensing domain like; TaF: Thumb and forefinger domain (blue); Csol: central solenoid (green); CTD: C-terminal domain (brown). Movements of the S6ext correspond to a bending with Gly4941 as a hinge point. These movements are amplified further away from the hinge point (yellow arrows). (**B**) A similar view of chains D, showing that the more subtle movements in the S6ext also result in smaller movements further away in the Csol. The further downstream movements that involve the remainder of the cytosolic shell are shown in Fig. 5E,F.

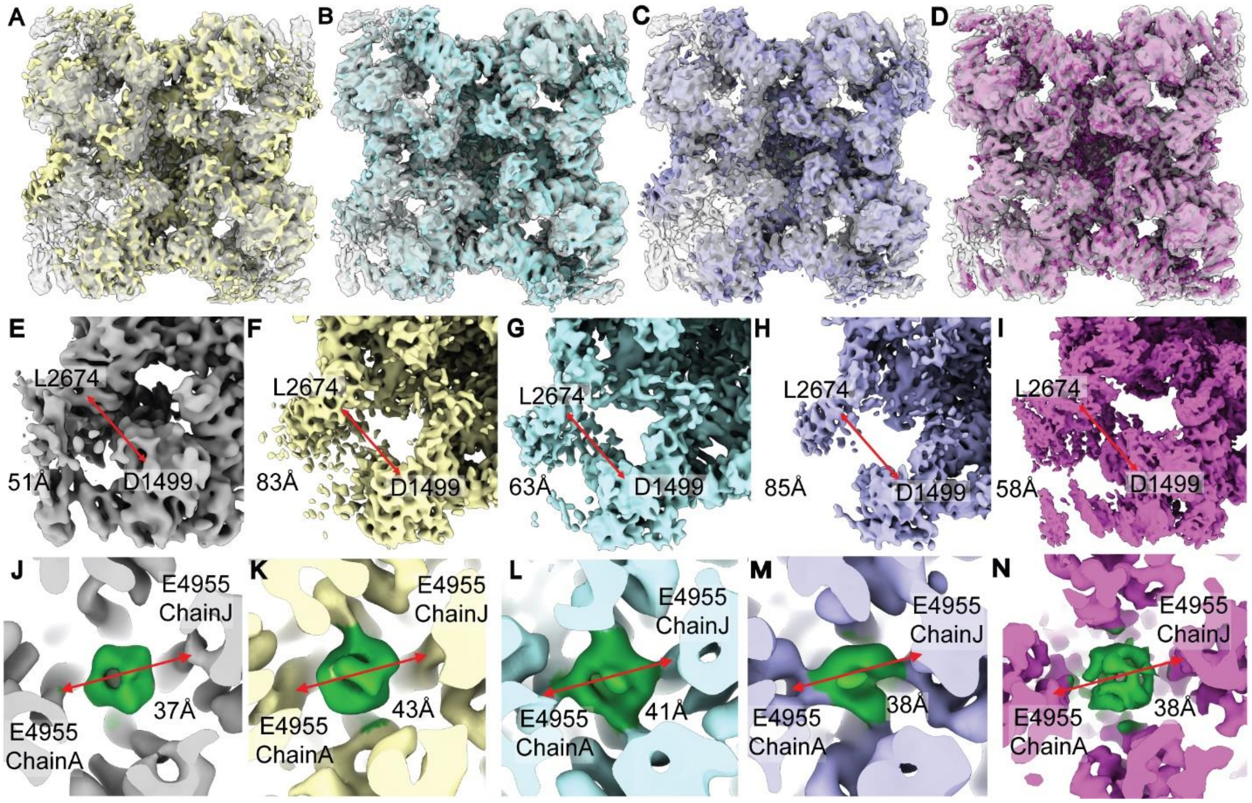

Fig S9. Comparisons of asymmetry various datasets. (A) – (D) Overall superpositions of the maps obtained from datasets 1 (EGTA condition, gray), dataset 2 (Ca^{2+} only; class 1: yellow; class 2: cyan; class 3: blue) and dataset 3 (global refined map, magenta). The EGTA map looks largely symmetrical despite being processed in C1. However, all other classes show significant degrees of asymmetry. The views are 'top' views facing the SR membrane. (**E**)-(**I**) Side by side views of the intersubunit area containing an asymmetric break. The colors as for panels A-D. To quantify the degree of asymmetry, the distance between two reference points is shown. These were obtained by rigid-body docking the Bsol and SPRY3 domains. There is a >30Å difference in this distance between the most symmetric (dataset 1) and most asymmetric classes (dataset 2, classes 1 and 3) (**J**)-(**N**) Side-by-side top views of the IpCa binding region, with density attributed to IpCa pseudocolored in green. The colors for the RyR1 densities are as in panels A-D. Distances are between the ends of the S6ext helices of diagonal pairs (corresponding to chains A an J in the global refined structure), measured via the C_α atoms of residue Glu4955. The S6/S6ext helix was fitted prior to the distance measurement. Although dataset 2 class 1 (panel K, yellow) shows a higher distance, smaller distances like this may be hard to interpret at the lower resolution (9 Å).

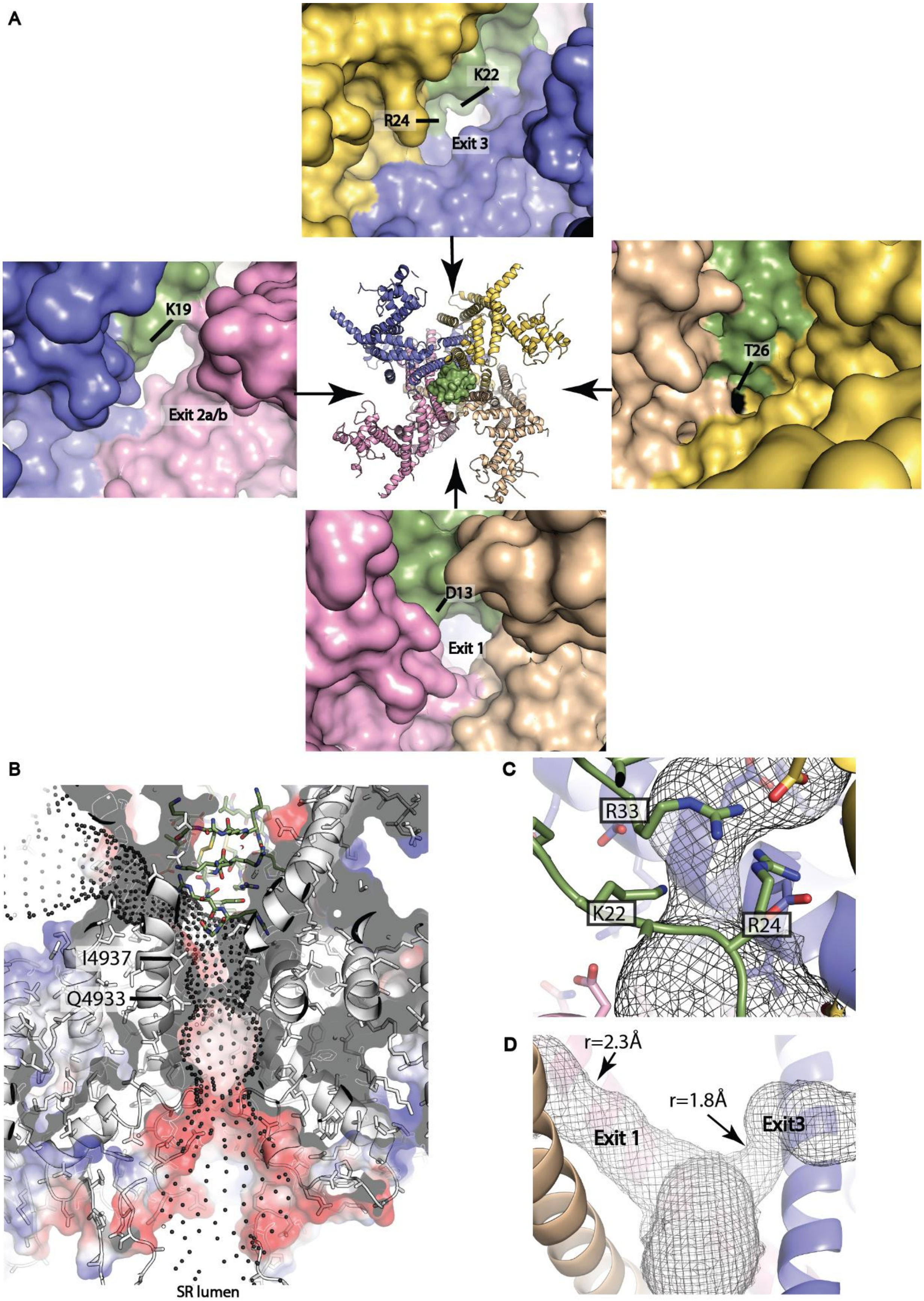
A
K22
R24
Exit 3
K19
Exit 2a/b
T26
D13
Exit 1
B
I4937
Q4933
SR lumen
C
R33
K22
R24
D
r=2.3Å
r=1.8Å
Exit 1
Exit3

Fig. S10. Permeation Pathways in IpCa-bound RyR1. (A) Surface representations of the S6ext helices, with each RyR1 subunit coloured differently. IpCa is shown in green surface. The center panel shows the views from which the surface representations are shown. The positions of select IpCa residues at the 4 sides are indicated for reference. The surface of Thr26 is shown in black. (**B)** HOLE plot showing the permeation pathway (delineated by black dots) from the SR lumen (bottom) towards IpCa (green sticks) and 'exit 1' (see Fig.7). RyR1 residues Ile4937 and Gln4933 in the permeation pathway are shown for reference. The colors represent the electrostatic surface potential of RyR1 (blue: positive; red: negative). (**C)** Caver tunnel (black mesh) around exit #3, showing how the narrowest part is surrounded by three positively charged residues of IpCa (labeled). (**D)** Comparison of tunnels (black mesh) for exits 1 and 3, as determined by Caver, with the radii of the narrowest parts of the tunnels indicated (arrows).

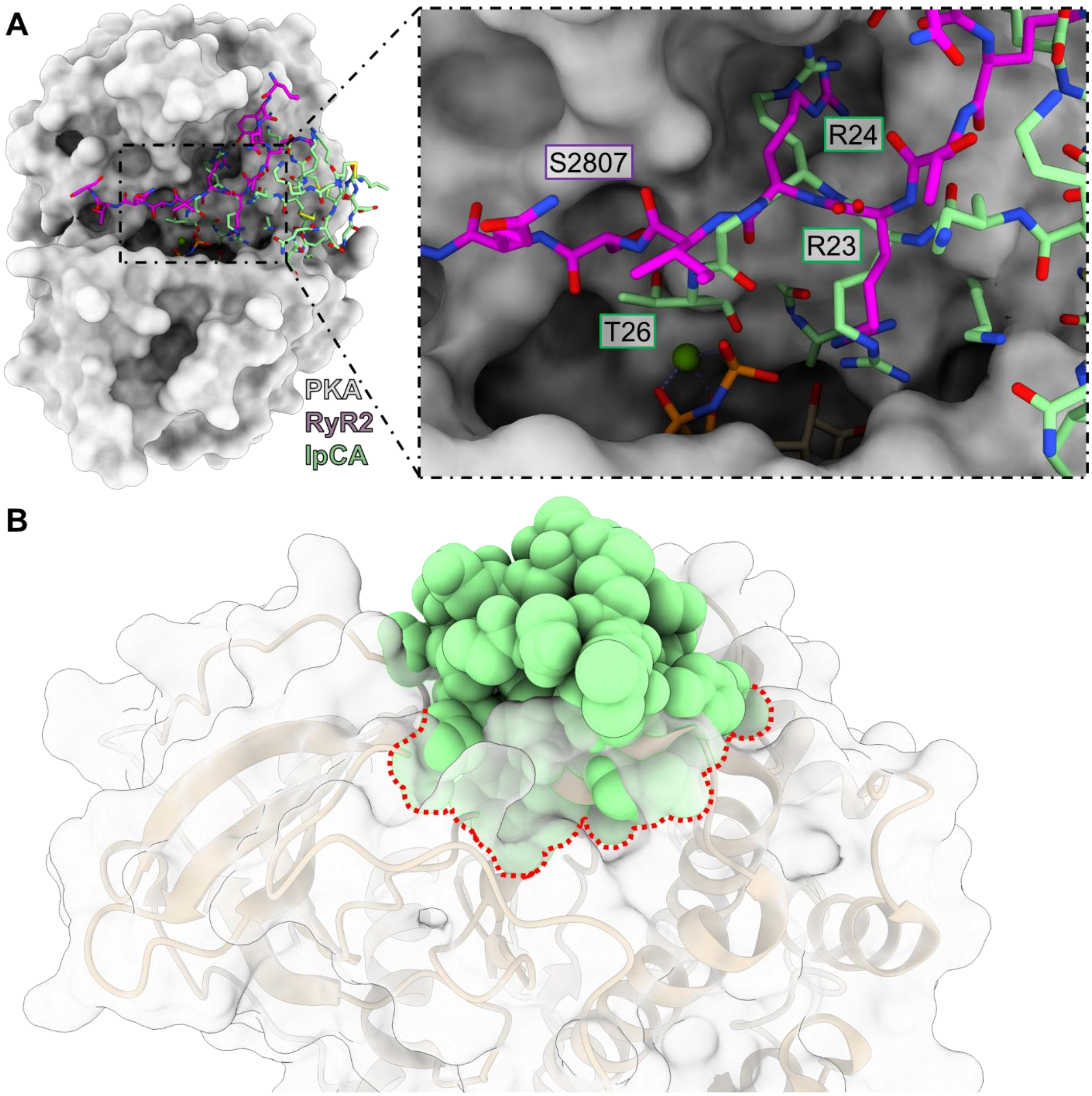

Fig. S11. IpCa in the active site of PKA. (**A**) superposition of IpCa (green sticks) to an RyR2 peptide (magenta sticks) bound to the PKA catalytic subunit (surface representation) (PDB: 6MM5). The superposition is based on the RRXS motif. **(B)** Same superposition as in panel A but showing the entire IpCa (green spheres) and the PKA (transparent white surface and wheat-colored cartoon). This shows that there would be large clashes between IpCa and the PKA (red dotted line indicates the steric clashes). Thus, the 22-30 loop containing Thr26 needs to adopt a very different conformation in order to be phosphorylated by PKA.

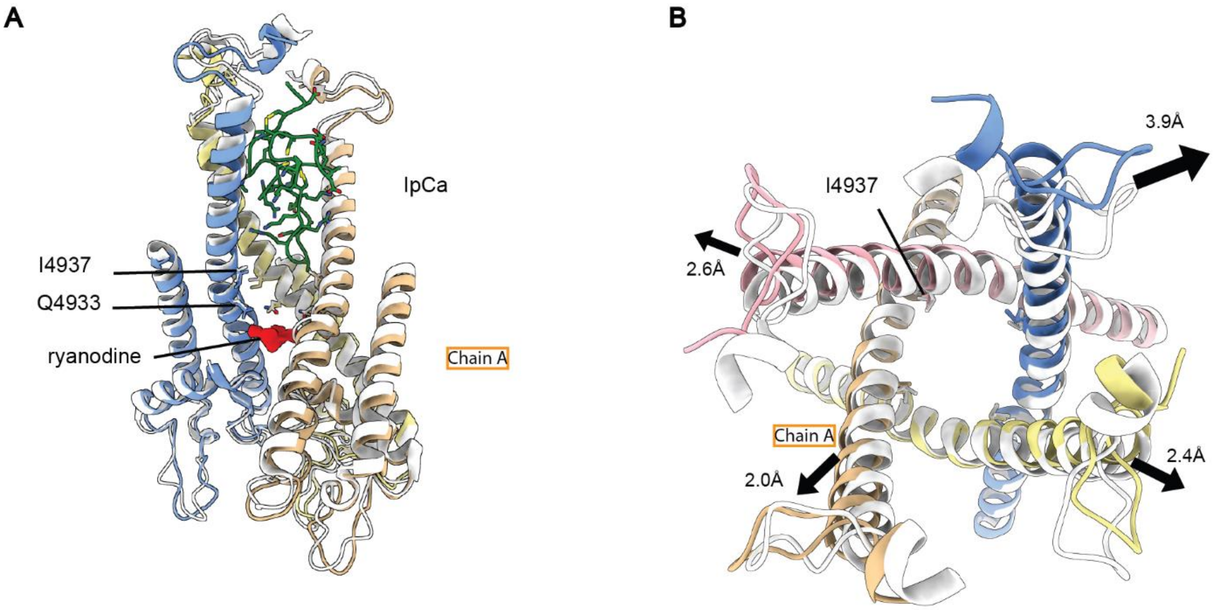

Fig. S12. Relative position of ryanodine and IpCa. (**A**) Superposition of the IpCa bound RyR1 (locally refined structure, dataset 3, colors) with a structure of RyR1 obtained in the presence of Ryanodine (PDB: 5TAW, white). Difference density, attributed to one or more molecules of ryanodine is shown in red (EMDB: 8387). For clarity, one subunit has been removed. Ryanodine binds within the transmembrane region, in contrast with IpCa (green), which binds in the cytosolic portion. The positions of Q4933 (likely interacting with ryanodine) and Ile4937 are indicated for reference. (**B**) Top view of the same superposition, showing the extra dilation for the S6ext helices. In both cases, the pore is open. The distances indicate the shifts at a reference point at the top of the S6ext helices (Cα atom for Glu4955). Note that the ryanodine-bound structure was solved with C4 symmetry, so any inherently asymmetric features are lost. The colors for the IpCa-bound structure are as for Figure 1.

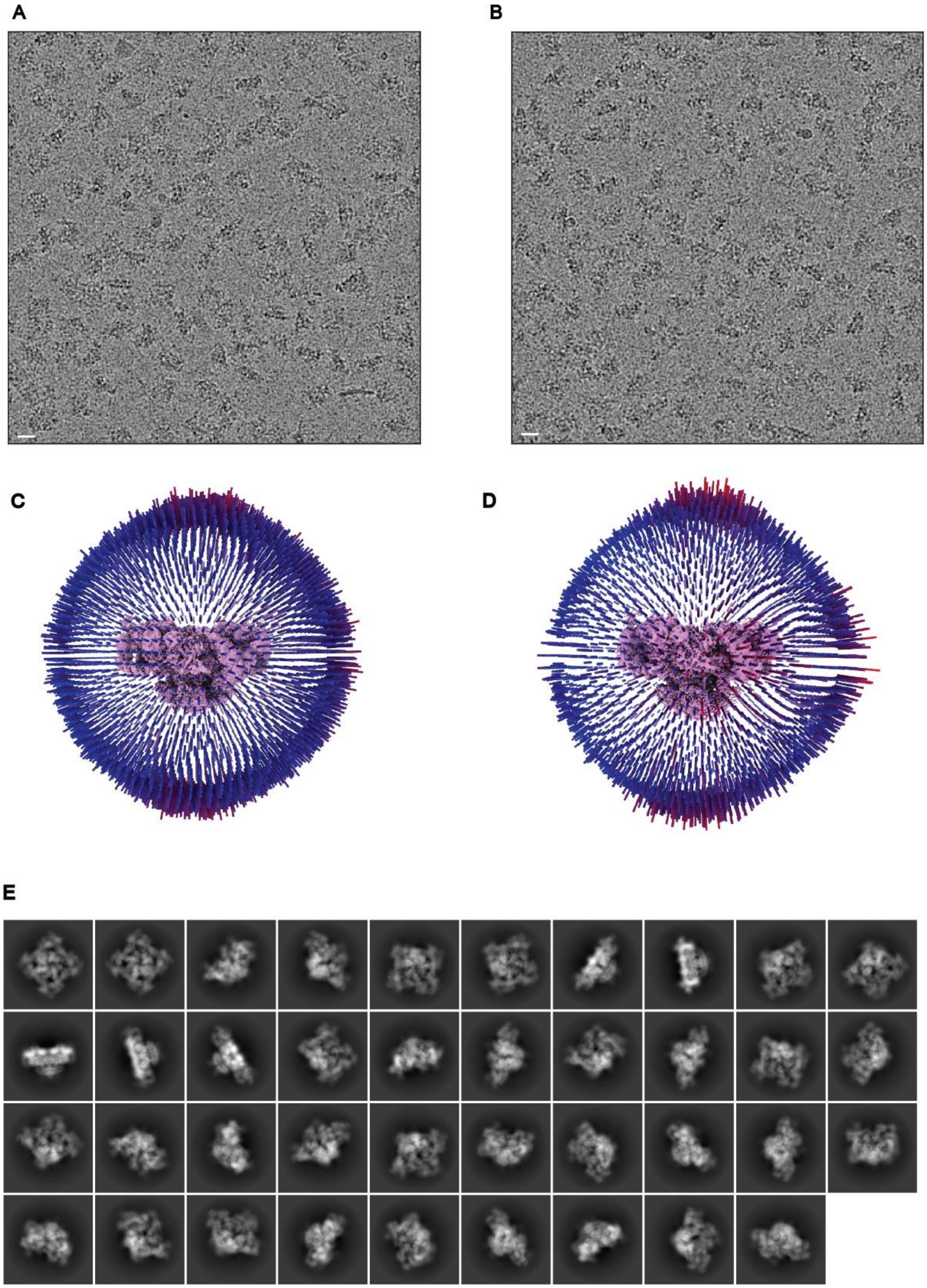

Fig. S13. Raw images and particle distributions. (A) Representative micrograph for dataset #2 (RyR1+IpCa in 30µM free Ca^{2+}). Scale bar (white) corresponds to 200Å. **(B)** Representative micrograph for dataset #3 (RyR1+IpCa+CaM_{1234}+ATP+Caffeine+ 30µM free Ca^{2+}). **(C)**,**(D)** Distribution plots for datasets #2 and #3, respectively, where the lengths of the cylinders correspond to the relative number of views. (**E**) 2D classification results from dataset #3.

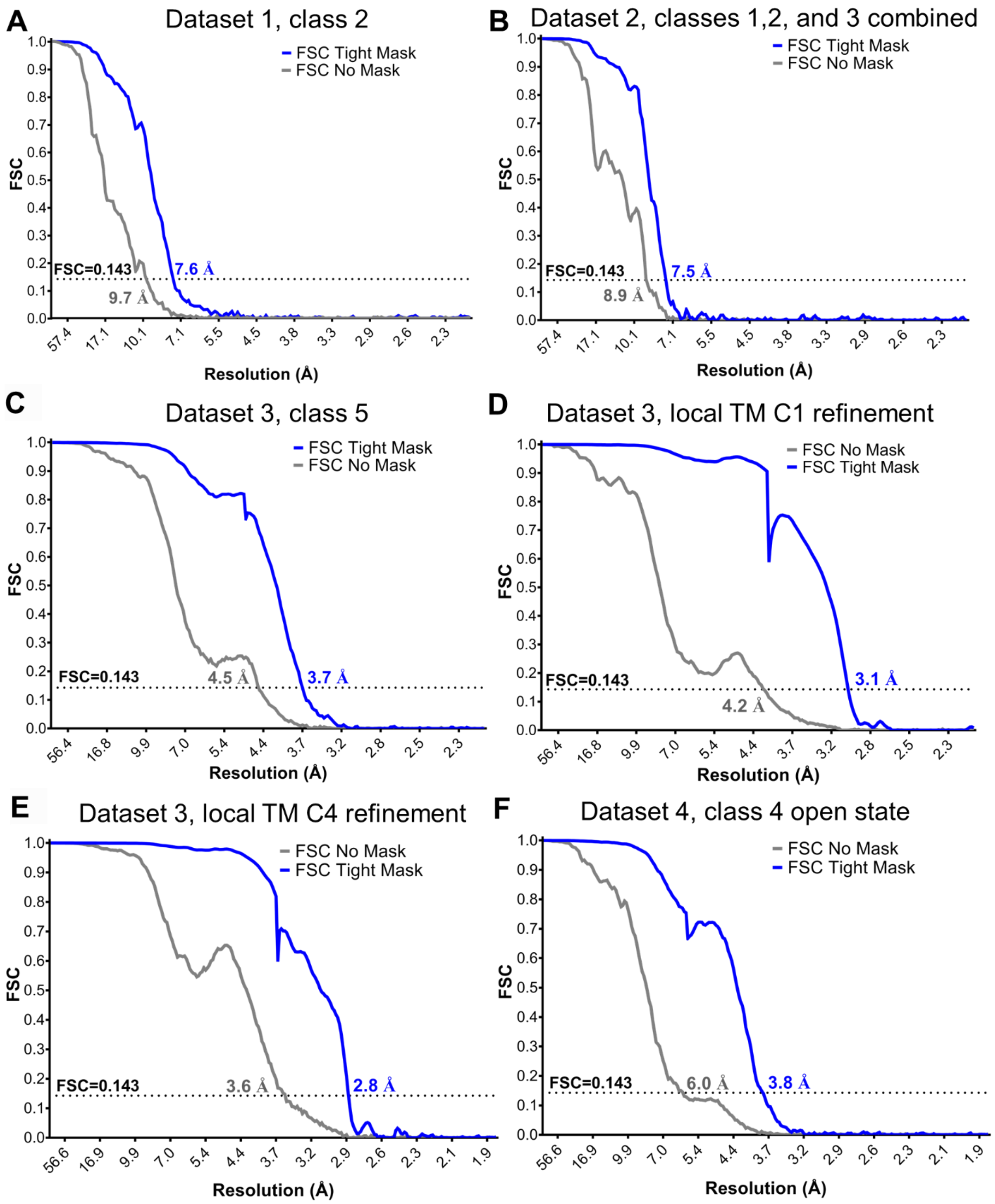

Fig. S14. Masked and unmasked Fourier Shell Correlation (FSC) curves. The curves were generated by by cryoSPARC v3.2 for **(A)** Dataset 1 (RyR + IpCa/EGTA) class 2, **(B)** Dataset 2 RyR1 + IpCa/30 µM Ca^{2+} classes 1,2 and 3 combined, **(C)** Dataset 3 (RyR1 + IpCa/Caff/ATP/CaM_{1234}/30 µM Ca^{2+}) class 5, **(D)** Dataset 3, classes 2,3, and 5 combined with particle extraction and local refinement with a TM mask, **(E)** Dataset 3, classes 2,3, and 5 combined with local TM mask refinement using C4 symmetry, and **(F)** Dataset 4, (RyR1+IpCa pT26/Caff/ATP/CaM_{1234}/30 µM Ca^{2+}) class 4 open state. Intercepts are shown at 0.143 FSC cut-offs.

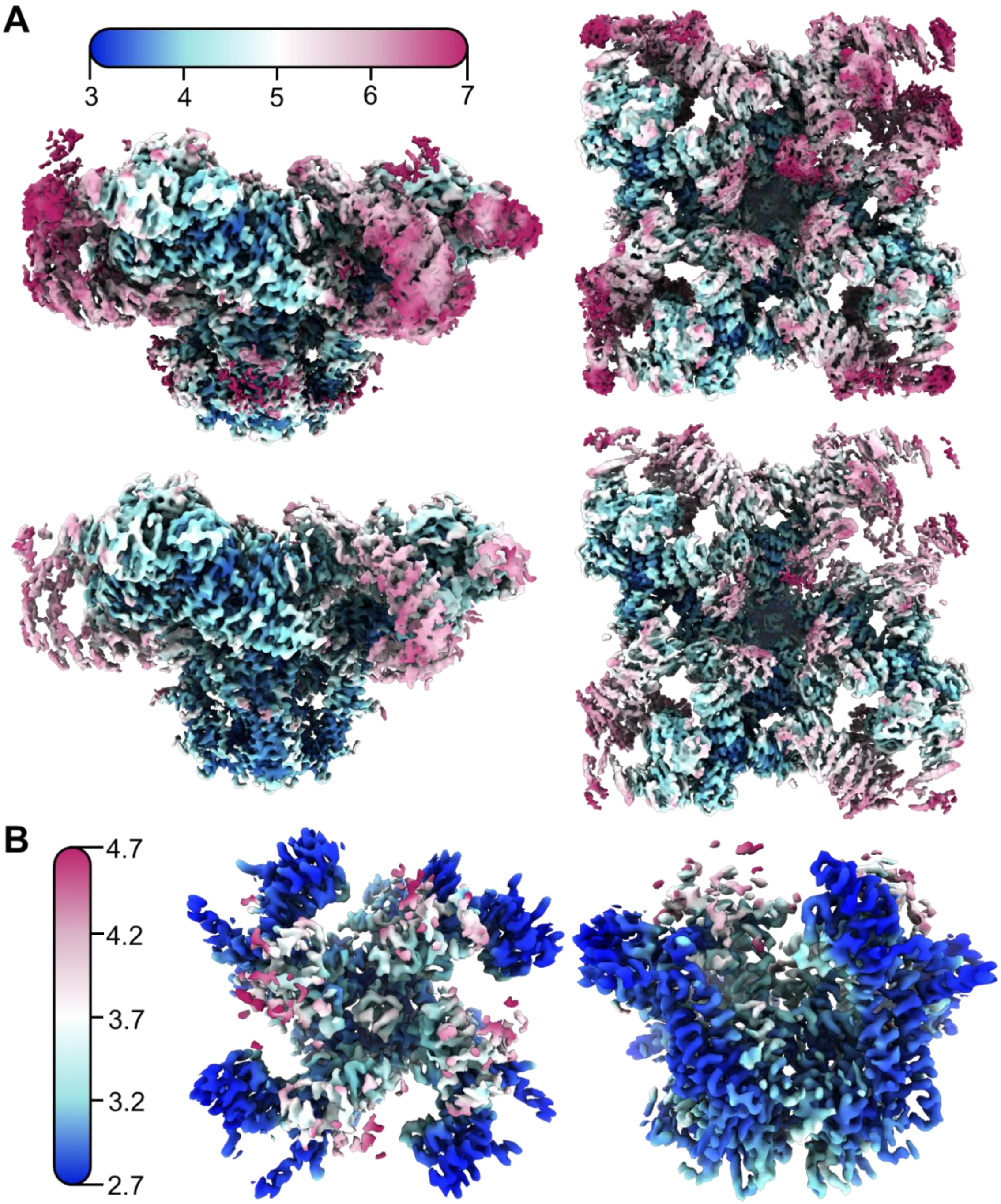

Fig. S15. Local resolution estimates. (A) Local resolution estimation coloured on global calcin-bound map (dataset 3, class 5) showing two different contour levels and views. (**B**) Local resolution estimation coloured on particle extracted and locally refined map **(**dataset 3) shown in two different views. Key colour bars are indicated for each panel with units in Angstrom (Å). The high local resolution for the VSD-like domains (dark blue) is likely the result of overfitting. However, using softer masks resulted in decreased density features for IpCa and the interacting helices.

Table S1.

Interactions between IpCa and RyR1. This table is meant only as a reference for potential H-bonds (HB) and salt bridges (SB), given limitations in resolution and potential dynamic changes.

IpCa		Rabbit RyR1			Distance (Å)	Type
residue	atom(s)	residue	Chain	atom		
R9	NH1	E4952	A	OE2	3.7	SB
C10	O	Q4949	A	NE2	3.4	HB
K11	NZ	Q4949	A	OE1	3.0	HB
K11	NZ	D4953	A	OD2	2.8	SB
K11	NZ	D4953	A	OD1	3.2	SB
R23	NH_2	D4945	A	OD2	2.4	SB
R23	NH_2	D4945	A	OD2	2.4	SB
T26	O	R4944	A	NH1	2.2	HB
K30	NZ	D4945	A	OD2	4.1	SB*
T26	N	D4938	D	OD2	2.7	HB
T26	OG1	D4938	D	OD2	2.4	HB
N27	N	D4938	D	OD2	3.5	HB
N27	ND2	D4938	D	O	3.1	HB
N27	ND2	E4942	D	OE1	3.2	HB
R24	NE	D4945	D	OD2	3.9	SB
R31	NE	E4942	D	OE2	2.9	SB
R31	NH1	E4942	D	OE1	2.4	SB
R31	NH2	E4942	D	OE2	2.6	SB
R33	NH1	D4945	D	OD2	3.0	SB
R33	NH2	D4945	D	OD1	3.2	SB
R33	NH2	D4945	D	OD2	2.5	SB
K20	NZ	D4945	J	OD1	3.5	SB
K20	NZ	E4948	J	OE2	3.8	SB
R24	NH1	D4938	J	OD2	2.5	SB
R24	NH1	D4938	J	OD1	3.7	SB
R24	NH2	D4938	J	OD2	3.6	SB
R24	NH2	D4938	J	OD1	3.7	SB
R24	NH2	D4938	J	OD2	3.6	SB
K19	O	Q4949	G	NE2	3.5	HB

Data collected using PDBePISA serever: https://www.ebi.ac.uk/pdbe/pisa/

* An additional potential ionic interaction between K30 and D4945 is also included

Table S2.

Enzyme kinetics. Michaelis-Menten Kinetic parameters determined using an ADP-Glo™ Kinase Assay. Readings are based on relative luminescence units (RLU) which are converted into moles of ATP hydrolyzed using an ADP-ATP standard curve according to manufacturer's protocol (see Methods). Kemptide sequence: LRRASLG.

Substrate	*K*m (μM)	*V*max ($\mu moles\ min^{-1} \times mg^{-1}$)	kcat (min^{-1})	kcat/*K*m ($min^{-1} \times \mu M^{-1}$)	Repeats
IpCa	22.0 ± 3.40	20.2 ± 0.93	672 ± 31.1	30.5	6
Kemptide	10.3 ± 2.16	8.34 ± 0.40	277 ± 13.2	27.7	6

Table S3.
Cryo-EM data collection, refinement and validation statistics

	**RyR1 + IpCa in activating condition (Global)** (EMD-27721) (PDB 8DUJ)	**RyR1 + IpCa particle subtraction + TM focused refinement** (EMDB-27695) (PDB 8DTB)	**RyR1 + IpCa TM focused refinement** (EMDB-27680) (PDB 8DRP)	**RyR1 + IpCa T26E in activating condition (Global)** (EMDB-27736) (PDB 8DVE)
**Data collection and processing**	Dataset 3, Class 5	Dataset 3, Classes 2,3,5	Dataset 3, Classes 2,3,5	Dataset 5, Class 4 (open class)
Magnification	75,000	75,000	75,000	130,000x
Voltage (kV)	300	300	300	300
Electron exposure (e–/Å^2)	50	50	50	50
Defocus range (μm)	– (1-3)	– (1-3)	– (1-3)	– (1-2.5)
Pixel size (Å)	1.07	1.07	1.07	0.94
Symmetry imposed	C1	C1	C4	C1
Initial particle images (no.)	763,000	763,000	763,000	693,000
Final particle images (no.)	144,529	322,724	322,724	41,834
Map resolution (Å)	3.74	3.05	2.83	3.84
FSC threshold	0.143	0.143	0.143	0.143
Map resolution range (Å)	3.0-7.5	2.7-5.0	2.7-4.5	3.2-8.0
**Refinement**				
Initial model used (PDB code)	6M2W	8DUJ	8DUJ	8DUJ
Model resolution (Å)	3.71	3.05	2.80	3.79
FSC threshold	0.143	0.143	0.143	0.143
Model resolution range (Å)	3.10-15.0	2.80-5.00	2.70-4.50	3.2-15.0
Map sharpening *B* factor (Å^2)	-103.6	-87.5	-96.7	-50.1
Model composition				
Non-hydrogen atoms	106,156	13,724	15,816	114,229
Protein residues	16,523	1,765	2,048	16,751
Ligands	188	166	184	188
B factors (Å^2)				
Protein	89.9	41.2	33.0	96.1
Ligand	66.4	74.7	58.6	110.8
R.m.s. deviations				
Bond lengths (Å)	0.004	0.004	0.005	0.004
Bond angles (°)	0.970	0.917	0.970	0.987
Validation				
MolProbity score	1.56	1.50	2.03	1.50
Clashscore	8.3	9.38	12.4	9.45
Poor rotamers (%)	1.25	0.99	3.22	0.63
Ramachandran plot				
Favored (%)	98.5	98.1	97.9	98.3
Allowed (%)	1.49	1.86	2.10	1.69
Disallowed (%)	0.01	0.00	0.00	0.02

Movie S1.
Morph comparing our structures of open RyR1 without and with IpCa bound. Only the transmembrane and adjacent region is shown. RyR1 subunits are shown in cartoon representation in four different colors. The IpCa shown at the end is shown in green spheres. The cytosolic extensions of the inner S6 helices (S6ext) need to move further apart to accommodate the IpCa.

Movie S2.
Morph comparing our structures of open RyR1 without and with IpCa bound, showing a 'top' view from the cytosol facing the SR membrane. RyR1 subunits are shown in cartoon in four different colors. Also shown are FKBP12.6 (red) and CaM_{1234} (purple). The morph shows the large asymmetric break, induced by IpCa binding, in the bottom left corner. The IpCa shown at the end of the animation is shown in green spheres.
